# High-level tumour methylation of *BRCA1* and *RAD51C* is required for homologous recombination deficiency in solid cancers

**DOI:** 10.1093/narcan/zcae033

**Published:** 2024-07-25

**Authors:** Lijun Xu, Brett Liddell, Ksenija Nesic, Franziska Geissler, Lauren M Ashwood, Matthew J Wakefield, Clare L Scott, Nicola Waddell, Olga Kondrashova

**Affiliations:** Cancer Research Program, QIMR Berghofer Medical Research Institute, Brisbane, QLD, Australia; The University of Queensland, Brisbane, QLD, Australia; Cancer Research Program, QIMR Berghofer Medical Research Institute, Brisbane, QLD, Australia; The University of Queensland, Brisbane, QLD, Australia; The Walter and Eliza Hall Institute of Medical Research, Melbourne, VIC, Australia; The Walter and Eliza Hall Institute of Medical Research, Melbourne, VIC, Australia; Cancer Research Program, QIMR Berghofer Medical Research Institute, Brisbane, QLD, Australia; The University of Queensland, Brisbane, QLD, Australia; The Walter and Eliza Hall Institute of Medical Research, Melbourne, VIC, Australia; Department of Obstetrics and Gynaecology, University of Melbourne, Melbourne, VIC, Australia; The Walter and Eliza Hall Institute of Medical Research, Melbourne, VIC, Australia; Department of Obstetrics and Gynaecology, University of Melbourne, Melbourne, VIC, Australia; Cancer Research Program, QIMR Berghofer Medical Research Institute, Brisbane, QLD, Australia; The University of Queensland, Brisbane, QLD, Australia; Cancer Research Program, QIMR Berghofer Medical Research Institute, Brisbane, QLD, Australia; The University of Queensland, Brisbane, QLD, Australia; The Walter and Eliza Hall Institute of Medical Research, Melbourne, VIC, Australia

## Abstract

In ovarian and breast cancer, promoter methylation of *BRCA1* or *RAD51C* is a promising biomarker for PARP inhibitor response, as high levels lead to homologous recombination deficiency (HRD). Yet the extent and role of such methylation in other cancers is not clear. This study comprehensively investigated promoter methylation of eight homologous recombination repair genes across 23 solid cancer types. Here, we showed that *BRCA1* methylated cancers were associated with reduced gene expression, loss of heterozygosity (LOH), *TP53* mutations and genomic features of HRD. We identified *BRCA1* methylation in 3% of the copy-number high subtype of endometrial cancer, and as a rare event in six other cancer types, including lung squamous cell, pancreatic, bladder and stomach cancer. *RAD51C* promoter methylation was widespread across multiple cancer types, but HRD features were only observed for cases which contained high-level tumour methylation and LOH of *RAD51C*. While *RAD51C* methylation was frequent in stomach adenocarcinoma (6%) and low-grade glioma (2.5%), it was mostly detected at a low tumour level, suggestive of heterozygous methylation, and was associated with CpG island methylator phenotype. Our findings indicate that high-level tumour methylation of *BRCA1* and *RAD51C* should be explored as a PARP inhibitor biomarker across multiple cancers.

## Graphical Abstract

**Graphical Abstract:**
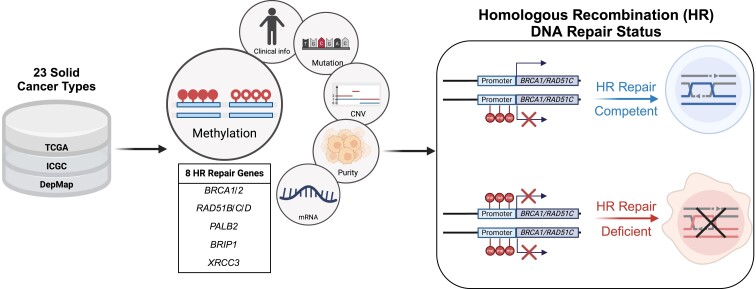

## Introduction

The homologous recombination repair (HRR) DNA double-strand break repair pathway can be disrupted in cancer, resulting in homologous recombination deficiency (HRD). HRD cancers tend to accumulate additional DNA damage, leading to specific genomic scarring patterns (1). HRD can be exploited therapeutically using platinum agents or a class of DNA repair inhibitors, called poly (ADP-ribose) polymerase inhibitors (PARPi), which have had profound impact in the clinic. The efficacy of PARPi therapy was initially demonstrated in high-grade serous ovarian cancer (referred to as OV hereafter) and breast cancer (BC), in which HRD was driven by germline pathogenic variants in either the *BRCA1* or *BRCA2* genes (2). However, a substantial proportion of cancers without *BRCA1/2* mutations can also manifest somatic mutational HRD molecular features, indicating the existence of other mechanisms of HRD. Biallelic inactivation of other core HRR genes including *RAD51C*, *RAD51D* (3) and *PALB2* have been associated with HRD (4), while alterations in *BRIP1* (5), *RAD51B* (6) and *XRCC3* (7) have also been suggested to impact the competency of the HRR pathway.

Promoter-associated DNA CpG methylation has been reported in cancer for some HRR genes, specifically, *BRCA1* and *RAD51C* (8–10). Homozygous promoter methylation of these genes (methylation of all gene copies) can result in silencing of the respective genes, leading to the HRD phenotype (11) and subsequently, susceptibility to PARPi therapy, as supported by pre-clinical and retrospective clinical cohort analyses (12). Promoter methylation of *BRCA1* and *RAD51C* in OV and BC has been extensively characterised (9,13). In OV, the prevalence of *BRCA1* methylation was reported in 15% of HRD cases and *RAD51C* methylation in 1% of HRD cases (13). A recent study used HRD-associated genomic patterns to provide evidence of HRD in 30 different cancer types, noting the presence of *BRCA1* and *RAD51C* methylation (14). Furthermore, evidence has linked abnormal methylation of *BRCA1* in normal tissue during early development stages (constitutional *BRCA1* promoter methylation) to an increased risk of OV and BC (15). However, despite the clinical relevance, characterisation of HRR-methylated cases in other cancer types and association with an HRD phenotype has not yet been explored.

In this study, we explore publicly available solid cancer datasets, including The Cancer Genome Atlas (TCGA) and International Cancer Genome Consortium (ICGC) (16), encompassing 23 major cancer types to identify cancers with aberrant somatic promoter methylation in HRR genes. We assess the molecular profiles of cases with *BRCA1* or *RAD51C* methylation by integrating methylation arrays, RNA sequencing and DNA sequencing to assess somatic copy number aberrations (CNAs), mutations and genomic ‘scarring’ predictive of HRD, as well as molecular and clinical subtypes. Finally, we estimate the methylation zygosity by adjusting for tumour purity and ploidy to predict gene silencing.

## Materials and methods

### Datasets

We used publicly available data from TCGA and ICGC. To ensure adequate sample size for prevalence assessment and downstream analysis, we chose 22 solid cancer types with at least 100 samples analysed using the Illumina Human Methylation 450 (HM450) array, including 7 625 primary cancer samples and 727 matched normal samples. We incorporated the ovarian dataset from ICGC (81 primary high-grade serous ovarian cancer samples and 81 matched normal samples) instead of TCGA OV, which was assayed with a platform of limited coverage across genome Illumina Human Methylation 27 (HM27). Additionally, 33 stomach adenocarcinoma (STAD) cell lines from the DepMap dataset were included in our analysis.

This project used publicly available datasets. The QIMR Berghofer Human Research Ethics Committee approved use of public data (P2095).

### Promoter methylation

For TCGA, the masked HM450 raw intensity files were downloaded from Genomic Data Commons (GDC) between April – June 2022 using GDC Data Transfer Tool Client (v1.6.0) (https://portal.gdc.cancer.gov/). For ICGC, the HM450 raw intensity files were accessed from the Gene Expression Omnibus (GEO) accession GSE65821 in March 2022. The raw HM450 data was converted into beta values (Supplementary Methods). For DepMap, reduced representation bisulfite sequencing data for 33 STAD cell-lines was obtained from Sequence Read Archive (SRA) under the accession number PRJNA523380 (accessed in April 2023), was processed and converted to percentage methylation per CpG site (Supplementary Methods).

A set of probes was selected to assess promoter methylation status, including CpG probes proximate to promoter regions or overlapping CpG islands with low median methylation levels (≤0.2) and low variability (IQR ≤ 0.2) (Supplementary Table S1 and Supplementary Figure S1). To reduce background noise, we evaluated the median methylation levels for eight HRR genes in the normal tissue across each cancer type. As such, *BRIP1* and *RAD51B* were excluded from breast cancer analysis due to high median methylation in normal tissue. Promoter methylation in primary tumours was assessed using at least 60% of selected probes, with methylation ≥0.25. Further details about probe selection and determination of the methylation status are provided in Supplementary Methods.

### Molecular characteristics

CpG island methylator phenotype (CIMP) classification was obtained from Yates et al., (2022) (17) for TCGA STAD, BC, low-grade glioma (LGG) and uterine corpus endometrial carcinoma (UCEC). For ICGC OV and TCGA testicular germ cell tumour (TGCT) dataset, we performed classification following the same methods (17).

Gene expression data for TCGA was obtained as pre-processed gene counts from GDC between April and June 2022 using the GDC Data Transfer Tool Client (v1.6.0) (https://portal.gdc.cancer.gov/). The ICGC and DepMap mRNA sequencing data were processed as described by Patch *et al.* (13). All gene counts data was filtered for lowly expressed genes and transformed to log2 counts-per-million (CPM) using edgeR (v3.40.2) (18).

Mutation data for TCGA was sourced from the Multi-Center Mutation Calling in Multiple Cancers project (MC3) (19). For ICGC, the method used to detect substitutions and indels is reported in Patch *et al.* (13). Somatic mutation for DepMap was obtained from the DepMap Portal (https://depmap.org/portal/; release 23Q2; accessed in Oct 2023).

CNAs for TCGA were obtained from GDC (June 2023). For ICGC, we used copy number segment information from whole genome sequencing (WGS) data using ascatNGS (v4.0.1) (20). For DepMap, the pre-processed copy number and LOH information was obtained from the CCLE ABSOLUTE (CCLE_ABSOLUTE_COMBINED_20 181 227, 2019 release).

HRD scores for TCGA were obtained from Knijnenburg *et al.* (6). For ICGC and DepMap, the HRD scores were calculated using scar HRD package (21) (v0.1.1).

Further details about determination of molecular characteristics are provided in Supplementary Methods.

### Methylation correction (purity and copy number)

Individual beta values for the selected probes as enlisted in the promoter methylation section were corrected using purity and gene-level copy number. We adopted the equation from VanLoo lab (22):


\begin{equation*}{{m}_b} = \frac{{\rho {{n}_t}{{m}_t} + {{n}_{n,i}}{{m}_{n,i}}\left( {1 - \rho } \right)}}{{\left( {\rho {{n}_t} + {{n}_{n,i}}\left( {1 - \rho } \right)} \right)}}\end{equation*}

$\rho \ = \ tumour\ purity;\ {{m}_b}\ = \ bulk\ tumour\ methylation\ rate$ ; ${{m}_t}\ = \ pure\ tumour\ methylation\ rate$; ${{m}_{n,i}}\ = \ normal\ methylation\ rate$; ${{n}_t}\ = \ tumour\ copy\ number$; ${{n}_{n,i}}\ = \ normal\ copy\ number.$

The normal copy number ${{n}_{n,i}}$was assumed to be 2 for all specimens. The normal methylation rate $\ ({{m}_{n,i}})$ was calculated as the mean beta value of the corresponding normal tissue within the same dataset. TCGA LGG did not have data available for matched normal tissue, therefore we used normal tissue from glioblastoma multiforme (GBM) instead.

### DepMap drug sensitivity

Drug sensitivity information (drug sensitivity replicate-level dose (CTD^2)) was obtained from the DepMap Portal (https://depmap.org/portal/; release 23Q2; accessed in October 2023). Drug sensitivity curve was generated using R package drc (v3.0-1) (23).

### Statistical tests

Fisher's exact test was performed to test the independence between the CIMP classification/other molecular subtypes and *BRCA1* or *RAD51C* methylation status. The comparison of HRD scores between groups was performed using the Mann–Whitney *U* test when the group number equals 2 or the Kruskal–Wallis test when the group number was >2. The posthoc Dunn's test corrected by Benjamini–Hochberg adjustment was performed following a significant Kruskal–Wallis test. *P*-value and *q*-value of <0.05 was considered statistically significant for statistical testing.

All other methods and sources for cancer subtypes, patient information and survival analysis are described in detail in the Supplementary Methods.

## Results

### Distinct promoter methylation landscape of *BRCA1* and *RAD51C* compared to other HRR genes

We evaluated the promoter methylation profile of eight HRR genes (*BRCA1*/*2*, *RAD51B*/*C*/*D*, *BRIP1*, *PALB2* and *XRCC3*), using data from TCGA and ICGC. Our analysis was focused on solid cancer types with methylation array data available for at least 100 samples (except for OV, *n* = 81 samples), resulting in 7619 primary cancer samples from 23 solid cancer types assessed.

Our focus was on the promoter regions, which were extended to include overlapping CpG islands and directly adjacent lowly methylated CpGs (Supplementary Table S1). Of the eight HRR genes examined, the median methylation observed at the promoter regions of *RAD51D* and *XRCC3* was ≤25% in all samples across all cancer types assessed (Supplementary Figures S2-S10), suggesting absence of promoter methylation for these two genes. Median methylation ≤25% was also found at the promoter regions of *BRIP1* and *BRCA2* for most cases, with only rare outliers. Notably, we observed one case of *BRCA2* promoter methylation in BC (Supplementary Figure S2 and S4). *BRIP1* promoter methylation (median value > 40%) was observed in one to two cases of cervical squamous cell carcinoma (CESC), liver hepatocellular carcinoma (LIHC), STAD and UCEC (Supplementary Figure S2 and S5).

**Figure 1.**
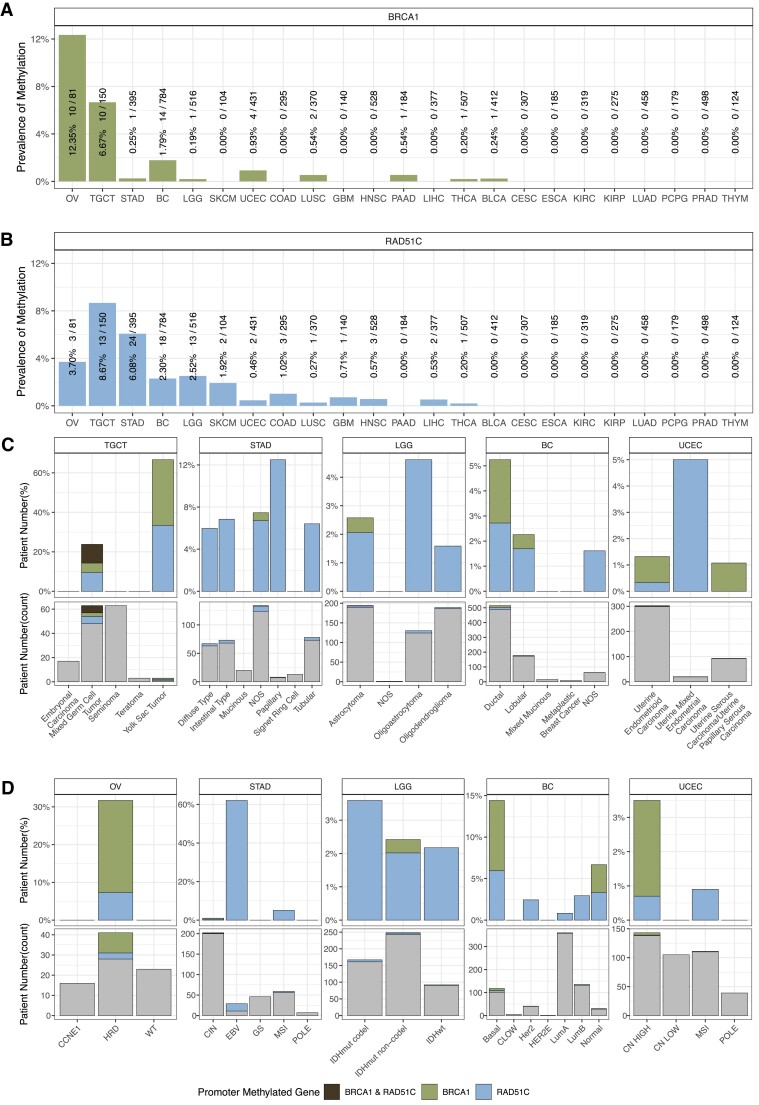
*RAD51C* and *BRCA1* promoter methylation across 23 solid cancer types. The prevalence of *BRCA1* (**A**) and *RAD51C* (**B**) promoter methylation in 23 cancer types, combining data from TCGA (22 cancer types) and ICGC (high-grade serous ovarian cancer). Cancer types are arranged by the highest combined prevalence of methylation (*meBRCA1* and *meRAD51C*). Promoter methylation status is determined based on specific CpG probes within 200 bp of the promoter region. The bar chart illustrates the percentage of cases with methylation. The percentage of cases with methylation, number of cases with methylation and total number of cases is indicated above each bar. *MeBRCA1* and *meRAD51C* distributions within cancer types with more than three detected cases are detailed in terms of distinct histological subtypes (**C**) and molecular subtypes (**D**). Testicular germ cell tumour (TGCT) is exempted from molecular subtype classification due to the absence of well-established molecular subtypes. Both histological subtypes and molecular subtypes are presented as two panels: an upper panel showing percentages of cases with *meBRCA1* and *meRAD51C* and a lower panel displaying counts. Bars are colour-coded: black (co-exist *meBRCA1* and *meRAD51C*), green (*meBRCA1*), blue (*meRAD51C*), grey (no promoter methylation detected in either gene). Abbreviations are high-grade serous ovarian cancer subtype (OV): CCNE1 amplification (CCNE1), homologous recombination deficiency (HRD) and homologous recombination wild-type (WT); stomach adenocarcinoma subtype (STAD): Epstein-Barr virus infected (EBV), chromosomal instability (CIN) and microsatellite instability (MSI); low grade adenocarcinoma subtype (LGG): *IDH* mutated with hemizygous codeletion of chromosome arms 1p and 19q (IDHmut codel), *IDH* mutated without codeletion (IDHmut non-codel) and *IDH* wild type (IDHwt); breast cancer (BC) subtype: basal, Claudin-low (CLOW), HER2 positive (Her2), HER2-enhanced (HER2E), luminal A (LumA), luminal B (LumB) and normal-like (Normal); uterine corpus endometrial carcinoma (UCEC) subtype: copy number high (CN HIGH), copy number low (CN LOW), microsatellite instability (MSI) and DNA polymerase epsilon mutation (POLE).

Promoter methylation analysis of *BRCA1* and *RAD51C* contrasted with that of the other genes described, revealing a more diverse methylation landscape, which formed the focus of exploration in this study. Analysing selected promoter probes of *BRCA1* and *RAD51C*, we identified promoter methylation of *BRCA1* (*meBRCA1*) or *RAD51C* (*meRAD51C*) (Supplementary Figure S2, S3 and S8) in 15 out of 23 cancer types (Figure 1A, B, Supplementary Table S2). *MeBRCA1* was present in 10 cancer types (Figure 1a) and was prevalent in OV (12.4%) and BC (1.8%), consistent with previous reports (14). We also observed *meBRCA1* in TGCT (6.7%), which has been previously reported by Shen *et al.* (24). *MeBRCA1* was found in 0.93% of the UCEC cohort, which to our knowledge has not been previously reported in the literature. The distribution of *meRAD51C* was different from that of *meBRCA1*. *MeRAD51C* was detected in 13 cancer types (Figure 1B), with the highest prevalence observed in TGCT (8.7%), followed by stomach adenocarcinoma (STAD) (6.1%), OV (3.7%), low grade glioma (LGG) (2.5%) and BC (2.3%). In the eight cancer types where *meBRCA1* and *meRAD51C* were observed (Figure 1a and b), these events were mutually exclusive in individual samples, except for TGCT (Supplementary Figure S11). Strikingly within TGCT, the methylation profiles among samples with *meBRCA1* or *meRAD51C* or combined *meBRCA1* and *meRAD51C* showed low methylation signal across all the *BRCA1*/*RAD51C* promoter probes examined (Supplementary Figure S11), suggesting a distinctive methylation pattern of these two genes in TGCT compared to the other cancer types.

To explore potential drivers of *meBRCA1* and *meRAD51C*, we assessed correlations with histological and molecular subtypes for cancer types with more than three cases of *meBRCA1* or *meRAD51C* (Supplementary Table S3). With the exception of TGCT, where *meRAD51C* was enriched in mixed germ cell subtype (Fisher's exact; *q*-value = 0.003), we found no evidence of enrichment of histological subtype within *meBRCA1* or *meRAD51C* samples (Figure 1C). Instead, we identified associations between *meBRCA1* or *meRAD51C* and molecular subtypes of OV, STAD and BC (Figure 1D). In OV, we found both *meBRCA1* and *meRAD51C* exclusively within the HRD molecular subtype, with *meBRCA1* significantly enriched (Fisher's exact; *q*-value = 0.006). In STAD, *meRAD51C* was strongly associated with the Epstein-Barr Virus (EBV) infection molecular signature (Fisher's exact; *q*-value < 0.0001), while the sole STAD case with *meBRCA1* was of the chromosomal instability (CIN) subtype. *MeBRCA1* BC was enriched in the basal subtype (Fisher's exact; *q*-value < 0.0001) whereas *meRAD51C* BC did not exhibit specific subtype enrichment. Despite lacking statistical significance, all four *meBRCA1* UCEC cases belonged to the p53-mutant copy number (CN) high subtype.

### Clinical features of *BRCA1* and *RAD51C* methylated cases

**Figure 2.**
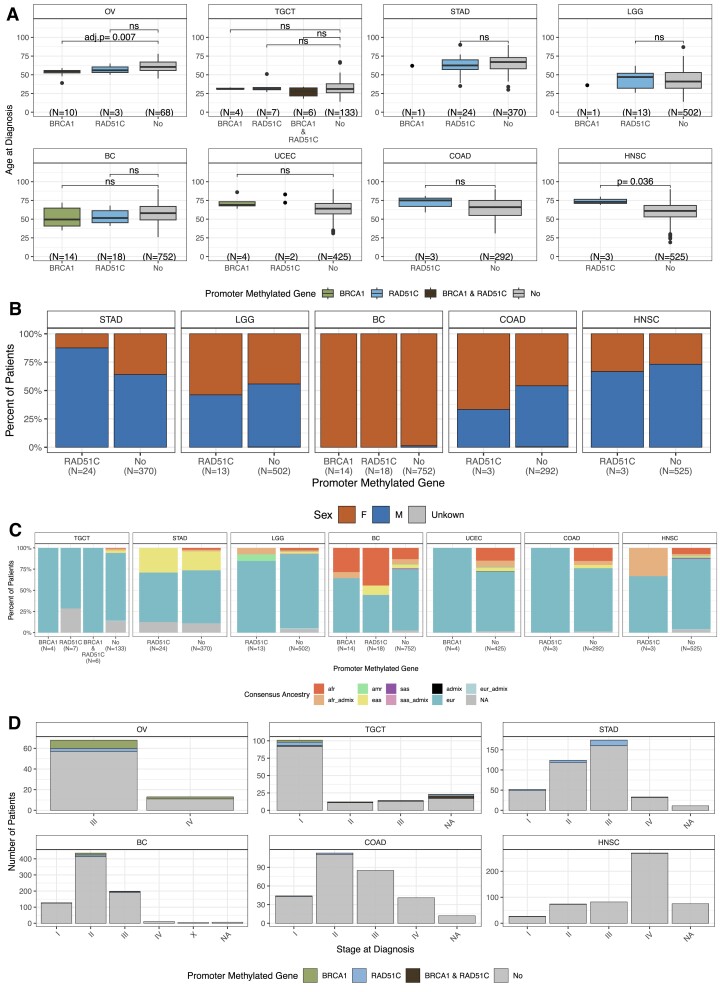
Clinical characteristics of patients with primary tumours exhibiting *BRCA1* and/or *RAD51C* promoter methylation. Only eight cancer types with more than two cases showing methylation in either *BRCA1* or *RAD51C* are included. Four characteristics were studied: (**A**) age, (**B**) sex, (**C**) ancestry and (**D**) stage. (A) Age distribution: boxplots show the distribution of age within a group. For groups with less than three samples, a dotplot is used. The boxplots are composed of the median (center bar) and IQR, with the whiskers extending to the maxima and minima, but no further than 1.5 × IQR. The outliers, defined as values beyond 1.5 × IQR, are represented by dots above or below box. A two-tail Wilcoxon test with Benjamini-Hochberg correction was used to compare between cancer types with at least three samples with *BRCA1* or *RAD51C* methylation. (B) Sex distribution: the percentage of patients identified as female (in orange) or male (in blue) is delineated based on the methylation status of *BRCA1* and *RAD51C*. TGCT, OV, UCEC, and CESC are excluded as they consist of only a single sex. (C) Ancestry distribution: the percentage of patients' ancestry, with colours indicating different ancestral origins as inferred from SNP arrays. The colour code is as follows: African (afr): orange, African admix (afr_admix): light orange, American (amr): green, East Asian (eas): yellow, European (eur): blue, European admix (eur_admix): light blue, South Asian (sas): purple, South Asian admix (SAS_admix): light purple, admix: black, missing information: grey. (D) Stage at diagnosis, LGG, UCEC and GBM are excluded as stage information is unavailable. Samples are grouped by stage and colours indicate the methylation status (*meBRCA1*: green, *meRAD51C*: blue, me*BRCA1&meRAD51C*: black, not methylated in either of the gene: grey). Abbreviations are high-grade serous ovarian cancer (OV), testicular germ cell tumour (TGCT), stomach adenocarcinoma (STAD), low-grade glioma (LGG), breast cancer (BC), uterine corpus endometrial carcinoma (UCEC), colon adenocarcinoma (COAD), head-neck squamous cell carcinoma (HNSC).

We next explored clinical features (age, sex, ancestry and tumour stage) and examined their association with *meBRCA1* and *meRAD51C*. We observed that patients with *meBRCA1* OV were marginally younger than those without *meBRCA1* and *meRAD51C* (Figure 2A, Supplementary Table S4, Two-tail Wilcoxon; *q*-value = 0.007). This association was confirmed in TCGA OV cohort (Supplementary Figure S12a, Two-tail Wilcoxon; *P*-value < 0.0001). Among patients diagnosed with STAD, we found that primary tumours from male patients were more likely to carry *meRAD51C* in comparison to those from female patients (Figure 2B, Supplementary Table S5, Fisher's exact; *P*-value = 0.02). We observed differences in ancestry distribution for BC, where methylated cases were more frequently in patients with African ancestry (*meBRCA1*: 28.5%, *meRAD51C*: 44.4%) compared to those with no promoter methylation (no methylation of either gene: 13.1%) (Figure 2C). As such, we observed that 10.9% of BC cases in patients with African ancestry have *BRCA1* or *RAD51C* methylation compared to 4.2% of all BC cases (without accounting for ancestry). This is consistent with the high prevalence of triple-negative breast cancer (TNBC) subtype in individuals with African ancestry (25) (Supplementary Figure S12b) and is of potential clinical importance due to worse outcomes associated with TNBC (26). In contrast, we found no enrichment of methylated cases by cancer stage, across any cancer type assessed (Figure 2D).

To further explore the clinical associations of *meBRCA1* and *meRAD51C*, we analysed the overall survival (OS) in the methylated and unmethylated cases (Supplementary Figure S13). Only subtypes enriched with *meBRCA1* or *meRAD51C* were included in this analysis to reduce the potential confounding effect from molecular or histological subtypes. We did not observe differences in OS between methylated and unmethylated cases across the evaluated cancer types—OV (HRD subtype), testicular mixed germ cell tumour, STAD (EBV subtype), LGG, BC (TNBC subtype) and UCEC (CN high subtype). However, due to the low methylated case numbers and diverse treatment and pre-treatment histories, this analysis was likely underpowered.

### Association of *BRCA1* and *RAD51C* methylation with global CpG island methylation patterns

**Figure 3.**
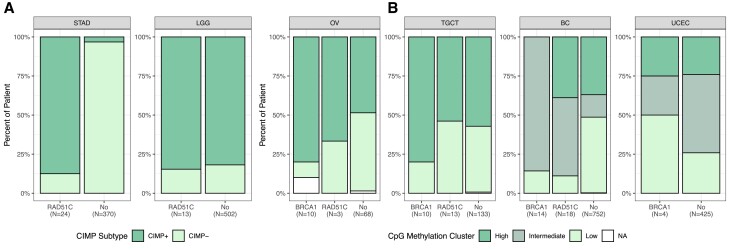
Global methylation and *BRCA1*/*RAD51C* promoter methylation. The prevalence of the CpG island methylator phenotype (CIMP) (**A**) or distinct global methylation clusters (**B**) in *meBRCA1* or *meRAD51C* samples in six cancer types. The cancer types with more than two cases methylated in either *BRCA1* or *RAD51C* are included. The percent of patients are categorised into dark green (CIMP phenotype or higher global CpG island methylation), green (intermediate global CpG island methylation), and light green (absence of CIMP phenotype or lower global CpG island methylation) within their respective primary cancer cohorts. Abbreviations are stomach adenocarcinoma (STAD), low-grade glioma (LGG), high-grade serous ovarian cancer (OV), testicular germ cell tumour (TGCT), breast cancer (BC), uterine corpus endometrial carcinoma (UCEC).

To investigate the potential connection between *meBRCA1* and *meRAD51C* and previously described global CpG island methylation patterns in cancer (17), we assessed methylation of CpG island-associated regions globally in each cancer case. CIMP is an epigenetic signature extensively explored in STAD (27) and LGG (28). The characterisation of this signature typically involves clustering of CpG-associated methylation probes (17,27,28). Using the clustering from Yates *et al.* (17), we found a strong enrichment of *meRAD51C* in CIMP+ samples in STAD, which were mostly EBV+ (Figure 3A, Supplementary Table S6, Fisher's exact; *q*-value < 0.0001). This association was absent in LGG, as the prevalence of CIMP+ samples in *meRAD51C* LGG (85%) closely mirrored that of the entire LGG cohort (82%).

While the presence of CIMP in OV, TGCT, BC and UCEC has not been described (17), subgroups with high global CpG island patterns of CpG methylation have been reported (17). In BC, both *meBRCA1* (Fisher's exact test; *q*-value < 0.0001) and *meRAD51C* (Fisher's exact test; *q*-value = 0.003) were found to be associated with intermediate levels of global CpG island methylation (Figure 3B). In contrast, we observed no statistically significant enrichment of specific global CpG island methylation profiles in *meBRCA1* or *meRAD51C* for OV, TGCT, and UCEC (Supplementary Table S6). Nonetheless, the majority of *meBRCA1* OV (89%), *meRAD51C* OV (67%) and *meBRCA1* TGCT (80%) exhibited relatively higher global CpG island methylation levels compared to the non-methylated samples within their respective cancer types (Figure 3B).

### Frequently co-mutated genes in methylated cases

To determine if me*BRCA1* and me*RAD51C* is directly associated with somatic mutation of cancer driving genes, we explored which genes were co-mutated with *meBRCA1* and *meRAD51C*. We compared the frequency of non-synonymous coding mutations in 1 025 cancer-associated genes from OncoKB (29,30) between methylated and unmethylated cases (Supplementary Table S7). By examining aggregated mutation frequencies across all cancer types, we found that *TP53* mutations were more prevalent across all cancer types in the *meBRCA1* subgroup, with 66% of methylated cases harbouring a *TP53* mutation compared to 43% of unmethylated cases (Supplementary Figure S14a). *TP53* mutations were found to be less frequent in the *meRAD51C* subgroup (33%) compared with the unmethylated subgroup (43%) across all cancer types (Supplementary Figure S14b). However, across all cancer types no other mutated genes were found to be significantly enriched in either the *meBRCA1* or *meRAD51C* subgroups.

**Figure 4.**
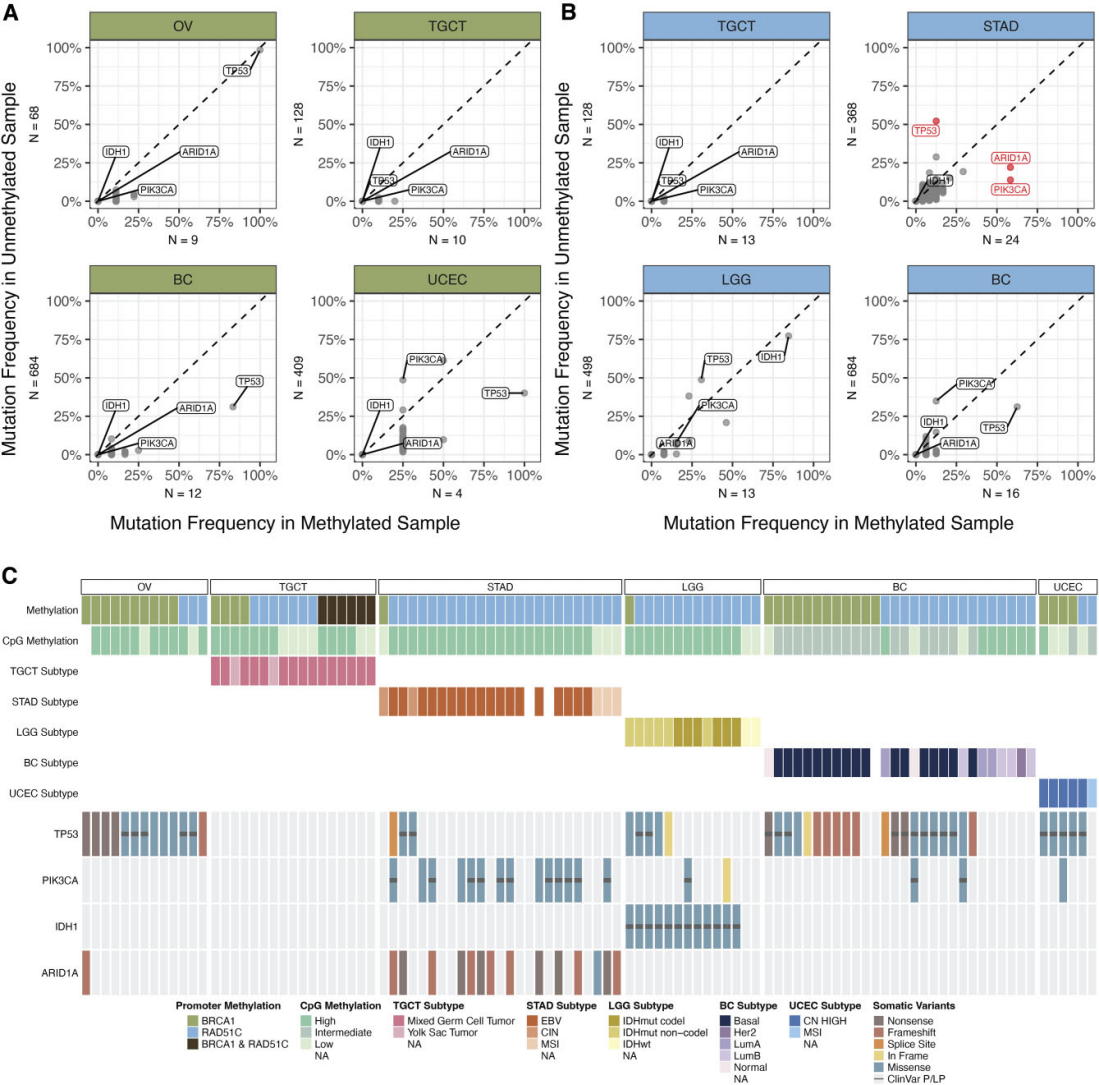
A comparison of somatic mutation frequencies in 1 025 cancer genes between samples with no methylation or *BRCA1/RAD51C* promoter methylation. (**A**) The frequency of somatic non-silent mutations in 1 025 cancer genes (OncoKB™) in samples with *BRCA1* promoter methylation (x-axis) and samples lacking promoter methylation (y-axis). Cancer types with more than three cases of methylated *BRCA1* are shown. (**B**) The frequency of somatic non-silent mutations in 1 025 cancer genes (OncoKB™) in samples with *RAD51C* promoter methylation (x-axis) and samples lacking promoter methylation (y-axis). Cancer types with more than three cases of methylated *RAD51C* are shown. Each data point is colour-coded based on its p-value from Fisher's exact test, adjusted using the Benjamini-Hochberg correction. Red data points indicate q-values < 0.05, signifying statistical significance, while grey data points indicate no significance. (**C**) The cancer subtype and somatic non-synonymous coding variants of *TP53*, *PIK3CA*, *IDH1* and *ARID1A* in *BRCA1*/*RAD51C* methylated cases. Cases are grouped into six cancer types, each bar shows profile of one case. Cases are ordered by their status of promoter methylation of *BRCA1* (green), *RAD51C* (blue) or co-occurring *BRCA1* and *RAD51C* (black). The global CpG methylation clustering of each case within their cancer types are colour coded into high (dark green), intermediate (green) and low (light green). Cancer subtypes of testicular germ cell carcinoma (TGCT), stomach adenocarcinoma (STAD) and low-grade glioma (LGG), breast cancer (BC) and uterine corpus endometrial carcinoma (UCEC) are colour coded for each cases accordingly. The subsequent coloured boxes indicate the presence or absence of somatic non-synonymous coding variants of *TP53*, *PIK3CA*, *IDH1* and *ARID1A*. Colour of the boxes corresponding to the types of non-silent somatic variants predicted, while black lines represent pathogenic (P)/likely pathogenic (LP) variants from ClinVar. STAD subtypes: Epstein-Barr virus infected (EBV), chromosomal instability (CIN), and microsatellite instability (MSI). LGG subtypes: *IDH* mutated with hemizygous codeletion of chromosome arms 1p and 19q (IDHmut codel), *IDH* mutated without codeletion (IDHmut non-codel), and *IDH* wild type (IDHwt). BC subtypes: basal, HER2 positive (Her2), luminal A (LumA), luminal B, and normal-like (Normal). UCEC subtypes: copy number high (CN HIGH) and microsatellite instability (MSI).

To determine if mutations were associated with *meBRCA1* or *meRAD51C* within specific cancer types, we narrowed our analysis to the six cancer types with at least four cases of *meBRCA1* or *meRAD51C*. *TP53* mutations were detected in all cases of OV and UCEC cancers with *meBRCA1*, as well as in most cases (83%) of *meBRCA1* BC (Figure 4A), however enrichment was not significant, likely due to small sample number. In contrast, *TP53* mutations were significantly less frequent in the *meRAD51C* STAD cases compared to the unmethylated cases (Figure 4B, 12.5% vs. 52.2%; Fisher's exact; *q*-value = 0.049). In LGG and BC, the prevalence of *TP53* variants was similar between the *meRAD51C* and unmethylated groups (Figure 4B). *TP53* variants were not detected in the TGCT cohort, regardless of *BRCA1* and *RAD51C* status.

For the *meRAD51C* STAD cases, *PIK3CA* and *ARID1A* mutations were significantly enriched compared to unmethylated samples (Figure 4B, Fisher's exact; *q*-value = 0.001 and 0.049, respectively). Corroborating the previous observation that *RAD51C* is not differently expressed between *IDH1* mutant LGG and *IDH1* wildtype LGG (31), *IDH1* variants were found at similar high frequency between me*RAD51C* group (85%) and the unmethylated group (77%). In addition, we found *IDH1* mutants to be exclusive to LGG in the me*RAD51C* cases across all cancer types.

To summarise, within BC, we observed an association of *meBRCA1*/*meRAD51C* with intermediate global CpG island methylation patterns, basal subtype, and the presence of *TP53* mutations (Figure 4C). In the case of STAD, our findings corroborate previous reports (27), with the co-occurrence of the CIMP subtype (high methylation), EBV infection, and *PIK3CA* mutations in *meRAD51C* samples (Figure 4C).

### Cancers with *BRCA1*, *RAD51C*, *BRCA2* or *BRIP1* promoter methylation displayed reduced expression of the relevant methylated gene

**Figure 5.**
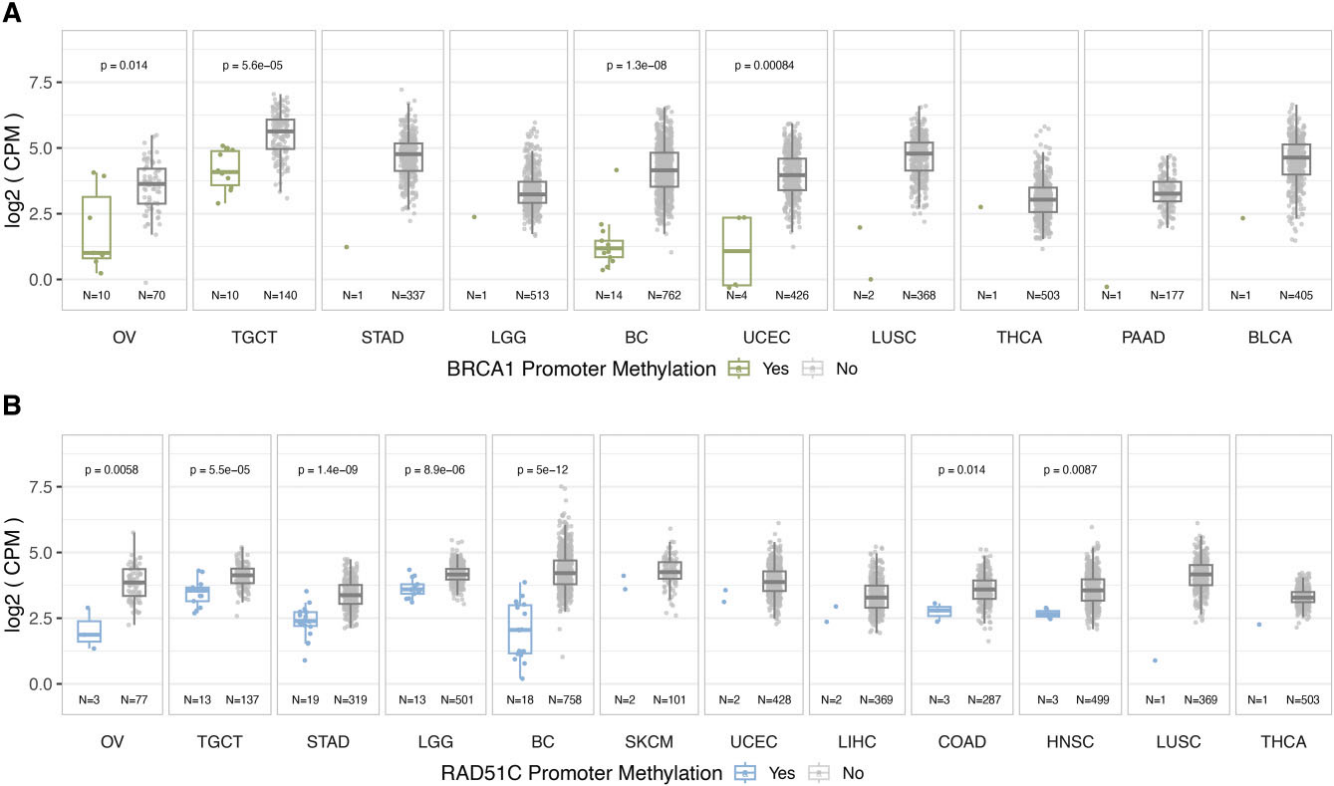
*BRCA1* and *RAD51C* promoter methylation associated gene expression. Gene expression of *BRCA1* (**A**) and *RAD51C* (**B**) in samples grouped by the presence of promoter methylation. Each box plot is a cancer type with normalised mRNA gene expression (log_2_ CPM) on y-axis and samples on x-axis grouped by gene promoter methylation status. The boxplots are composed of the median (center bar) and IQR, with the whiskers extending to the maxima and minima, but no further than 1.5 × IQR. Outliers, defined as values beyond 1.5 × IQR, are not shown in the boxplot. All data points are depicted with dots overlaid on the boxplot. Significant gene expression differences between methylated and unmethylated samples are shown with a two-tailed Wilcoxon signed-rank test. Abbreviations: high-grade serous ovarian cancer (OV), testicular germ cell tumour (TGCT), stomach adenocarcinoma (STAD), low-grade glioma (LGG), breast cancer (BC), uterine corpus endometrial carcinoma (UCEC), lung squamous carcinoma (LUSC), thyroid cancer (THCA), pancreatic adenocarcinoma (PAAD), bladder urothelial carcinoma (BLCA), skin cutaneous melanoma (SKCM), colon adenocarcinoma (COAD), liver hepatocellular carcinoma (LIHC).

We assessed if HRR gene promoter methylation was associated with reduced expression of the corresponding HRR gene, using matched RNA-seq data from individual cases. Significantly reduced gene expression was observed for *meBRCA1* (Figure 5A) and *meRAD51C* (Figure 5B) cases in the cancer types with at least three methylated cases. For the cancer types with lower numbers of methylated cases, the methylated cases showed a gene expression level that was below the median of the unmethylated cases. Similarly, gene expression was below the lowest quartile of the unmethylated cases for *BRIP1* (Supplementary Figure S15a) and *BRCA2* (Supplementary Figure S15b) methylated cases, with the exception of me*BRIP1* STAD case.

### *BRCA1* methylation associated with HRD scarring, while the impacts of *RAD51C* methylation varied

Having identified cases with promoter methylation and reduced corresponding gene expression in *BRCA1*, *RAD51C*, *BRCA2* and *BRIP1*, we proceeded to examine the correlation between promoter methylation levels and genomic HRD biomarkers, specifically the genomic scarring (HRD score) (1,32) and COSMICv2 Signature 3 (33). HRD scores were established and validated for application in OV and BC (32), and are calculated as a sum of three components: loss of heterozygosity (LOH), telomeric allelic imbalance (TAI) and large-scale state transition (LST).

**Figure 6.**
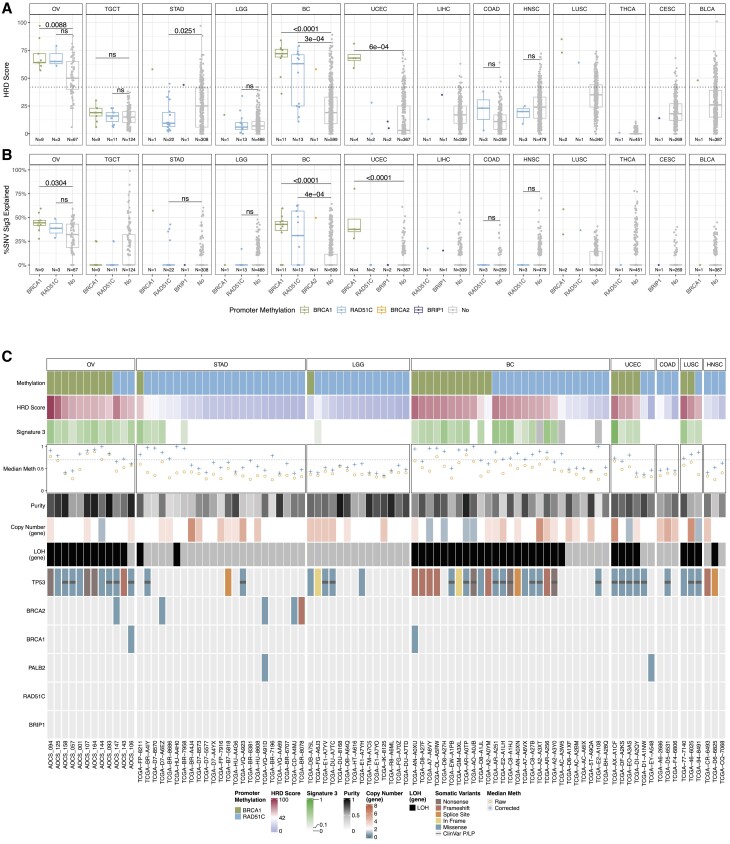
Molecular features of homologous recombination deficiency (HRD) in cases with promoter methylation of homologous recombination repair (HRR) genes. (**A**) Distribution of HRD scores and (**B**) proportion of single nucleotide substitutions explained by COSMICv2 signature 3 grouped by the promoter methylation status in *BRCA1*, *RAD51C*, *BRCA2*, *BRIP1*, *PALB2* (x-axis). Each box plot is a cancer type with HRD scores or Signature 3. A two-tailed Wilcoxon signed-rank test with Benjamini-Hochberg correction showed differences in HRD score or Signature 3 distribution in cancer types with at least three methylated samples. (**C**) The plot details various factors, including promoter methylation status of *BRCA1* (green) and *RAD51C* (blue), HRD score, COSMICv2 signature 3 contribution, of the selected promoter CpG probes in cases with *BRCA1* and *RAD51C* promoter methylation (raw: orange circle, corrected by purity and copy number: blue cross). The plot includes only cancer types with cases showing promoter methylation in either of the gene, however TGCT is excluded from this analysis due to the unavailability of methylation information for the matched normal tissue. The threshold for high methylation (median beta value of 0.7 post correction) is indicated with dashed line. Subsequent rows provide information on the estimated tumour purity, copy number status for the corresponding methylated gene, and the presence of loss of heterozygosity (LOH) for the corresponding methylated gene. The oncoplot shows the presence of somatic mutations in HRR genes with variant annotation of pathogenic (P) or likely pathogenic (LP) from ClinVar. Samples are ordered by HRD scores. Abbreviations: high-grade serous ovarian cancer (OV), testicular germ cell tumour (TGCT), stomach adenocarcinoma (STAD), low-grade glioma (LGG), breast cancer (BC), uterine corpus endometrial carcinoma (UCEC), glioblastoma multiforme (GBM), liver hepatocellular carcinoma (LIHC), colon adenocarcinoma (COAD), head-neck squamous cell carcinoma (HNSC), lung squamous carcinoma (LUSC), thyroid cancer (THCA), cervical squamous cell carcinoma (CESC), bladder urothelial carcinoma (BLCA).

In samples with *meBRCA1*, we observed higher HRD scores (Figure 6A) and/or Signature 3 (Figure 6B) across OV, BC, UCEC, LUSC and bladder urothelial carcinoma (BLCA), when compared to cases without promoter methylation (Supplementary Table S8). Increased HRD scores and Signature 3 were also found in all *meRAD51C* OV and the single LUSC case, as well as a subset of BC and STAD cases. Within the HRR genes other than *BRCA1* and *RAD51C*, we found elevated HRD scores and/or proportion of Signature 3 in the single *BRCA2*-methylated BC, *PALB2*-methylated LUSC and *BRIP1*-methylated LIHC cases, but not in the two *BRIP1*-methylated UCEC cases (Figure 6A, B). A similar trend was also observed when we reviewed the three individual components of HRD scores (Supplementary Figure S16).

To determine tumour-specific levels of *BRCA1* promoter methylation within samples, we corrected the methylation level by tumour purity and *BRCA1* gene-level copy number. We identified a subset of OV (e.g. AOCS-158, AOCS-057), BC (TCGA-D8-A1JL, TCGA-A2-A0YM, TCGA-D8-A27F and TCGA-EW-A1PB) and UCEC (TCGA-DF-A2KS, TCGA-D1-A1NW) cases that displayed relatively low tumour tissue levels of *BRCA1* methylation (Figure 6C) (<70% median methylation post-correction), suggesting potential subclonal or heterozygous methylation in these cases. Previous literature has described treatment driven methylation loss of *RAD51C* and *BRCA1* in patient-derived models (12,34,35). Therefore, we further investigated the treatment status of these *meBRCA1* cases with low tumour tissue levels of methylation. These cases were all primary surgical cases. One of the two OV samples (AOCS-057) had no history of chemotherapy for other malignancies, while AOCS-158 was exposed to neoadjuvant treatment prior to the sample collection (Supplementary Table S9). For TCGA, none of the low level me*BRCA1* samples reported history of other malignancy or exposure to neoadjuvant treatment. Of note, the primary samples for TCGA-DF-A2KS (UCEC) and TCGA-D8-A27F (BC) were collected on the same day as or prior to the diagnosis, which suggest a low possibility of other treatment exposure (Supplementary Table S9). We did not detect any variants likely to affect protein function in the HRR-related genes in these cases with low level me*BRCA1*, yet all these samples had high HRD scores suggestive of a HRD phenotype (≥42) (Figure 6C). These cases also had one or more copies of the *BRCA1* gene with evidence of LOH. This supported that both high and low levels of *BRCA1* methylation could be associated with HRD scarring in multiple cancer types, including OV, BC and UCEC, and could indicate a history of HRD. It also suggested that partial loss of methylation can occur during cancer development or early exposure to treatment.

For *meRAD51C*, we also identified a subset of cases with low tumour tissue levels of *meRAD51C* (defined as less than 70% median methylation post-correction). Most STAD (15 of 22) and all LGG cases showed low levels of methylation post-correction (Figure 6C), suggesting the methylation is heterozygous. These cases, as well as low-level *meRAD51C* colon adenocarcinoma (COAD) and HNSC, also mostly exhibited low HRD scores, absence of Signature 3 and absence of *RAD51C* LOH (Figure 6C). Additionally, unlike *meBRCA1*-low cases, BC and UCEC cases with low levels of *RAD51C* methylation, displayed HRD scores below the HRD threshold of 42 (TCGA-E2-A108, TCGA-D1-A1NW, TCGA-EY-A548) and lacked Signature 3. We should note, however, that a number of *meRAD51C* cases had low tumour purity, which may affect accurate determination of tumour methylation level. Additional complicating factors included the number of probes covering the *RAD51C* promoter being lower than for the *BRCA1* promoter (7 probes versus 21 probes), and the observed (Supplementary Figure S3, S8 and S11) and reported (34) heterogeneity for *meRAD51C* being higher.

### PARPi sensitivity in STAD with homozygous *RAD51C* methylation

**Figure 7.**
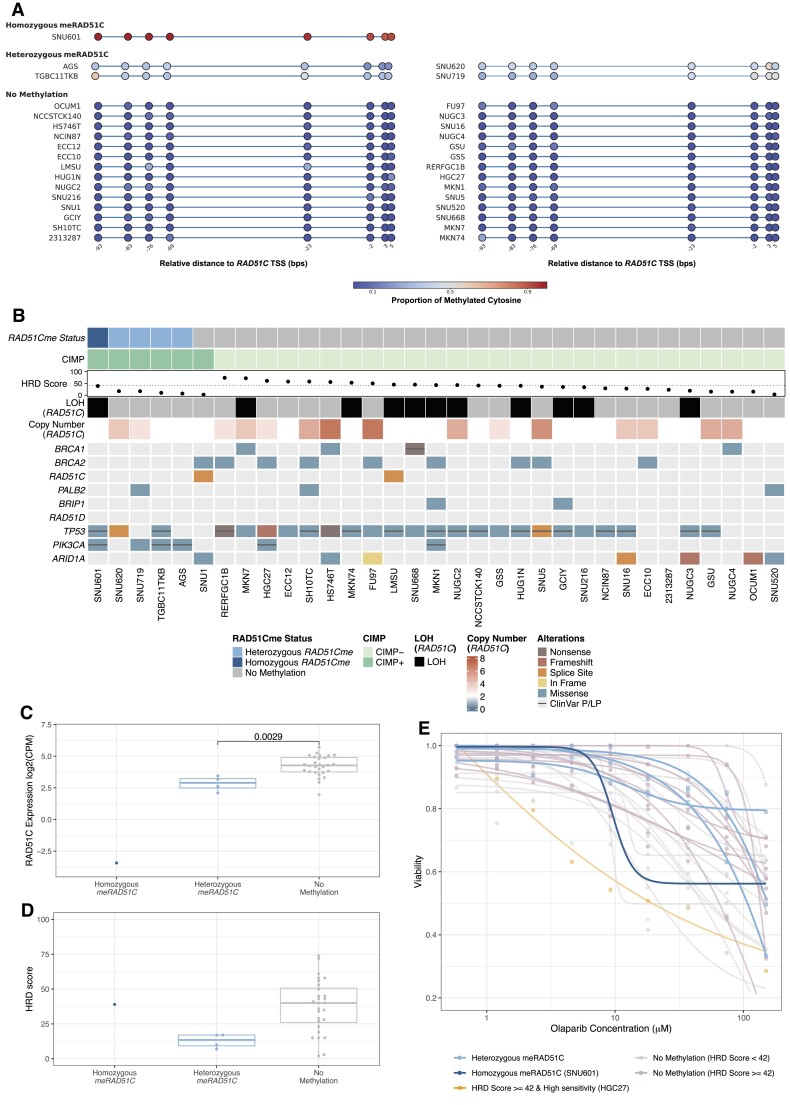
*RAD51C* promoter methylation, global CpG island methylation and homologous recombination deficiency (HRD) in stomach adenocarcinoma (STAD) cell-lines. (**A**) Methylation levels of eight individual CpG site within the *RAD51C* promoter region are represented on a colour scale from 0 (blue) to 1 (red) in 33 STAD cell-lines from DepMap. (**B**) An integrated summary showcases the *RAD51C* promoter methylation, global methylation (CIMP) status, HRD scores with the widely used threshold (in BC and OV) of 42 highlighted with dashed line. The presence of loss of heterozygosity (LOH) and copy number of *RAD51C* gene region are provided. The oncoplot shows the presence of somatic variants in HRR genes (*BRCA1*/*2*, *RAD51C*/*D*, *PALB2*, *BRIP1, PIK3CA*, *ARID1A* and *TP53*). Coloured boxes indicate types of non-silent somatic variants predicted by Depmap mutation pipeline, while black lines represent pathogenic (P) or likely pathogenic (LP) variants from ClinVar. (**C**) Normalised mRNA expression (log2 CPM) of *RAD51C* (y-axis) in samples grouped as homozygous (*N* = 1), heterozygous (*N* = 4) *RAD51C* promoter methylation (*meRAD51C*) and no methylation (*N* = 28) (x-axis). *P*-values shown from a two-tail Wilcoxon signed-rank test. (**D**) HRD scores (y-axis) in samples grouped as homozygous, heterozygous *meRAD51C* and cases with no *meRAD51C* detected (x-axis). (**E**) Olaparib sensitivity in 27 cell-lines (data unavailable for TGBC11TKB, RERFGC1B, GCIY, SNU520, NUGC2 and OCUM1). The changes of cell viability (y-axis) with Olaparib concentration (x-axis) are described with samples grouped primarily by *RAD51C* promoter methylation status (homozygous *RAD51C* promoter methylation: dark blue; heterozygous *RAD51C* promoter methylation: blue) and then HRD scores (no *RAD51C* promoter methylation and HRD score greater than or equal to 42: pink; no *RAD51C* promoter methylation and HRD score smaller than 42: grey).

Due to the high prevalence of *meRAD51C* in STAD being associated with low HRD scores, we wanted to further explore this event without the issue of contaminating stroma. To achieve this, we analysed an independent dataset of 33 STAD cell-lines from DepMap (36). We detected *meRAD51C* in five of the cell-lines (Figure 7A), with one of the five methylated cell-lines, SNU601, exhibiting a consistently high level of methylation across all CpG sites examined (termed homozygous *meRAD51C*). In contrast, the other four cell-lines displayed lower and heterogeneous methylation patterns, which we defined as heterozygous *meRAD51C* (Figure 7A). Interestingly, the SNU601 cell-line with homozygous *meRAD51C* was established from a STAD sample (ascites), from a patient who had received prior chemotherapy treatment, whereas the other methylated cell-lines were established from untreated primary cancer or metastatic lymph nodes (37,38). None of the methylated cell-lines showed evidence of truncating somatic variants in HRR genes (Figure 7B).

Consistent with findings in the TCGA STAD cohort, we observed that *meRAD51C* cell-lines exhibited a high global CpG island methylation pattern (CIMP subtype) and an enrichment of *PIK3CA* non-silent variants (Figure 7B & Supplementary Figure S17; CIMP Fisher's exact: *P*-value < 0.0001, *PIK3CA* Fisher's exact: *P*-value = 0.0017). However, none of the *meRAD51C* cell-lines carried somatic *ARID1A* variants, contrasting with the TCGA STAD cohort's results.

Heterozygous *meRAD51C* samples were associated with reduced *RAD51C* gene expression compared to those with no methylation (Figure 7C, two-tail Wilcoxon *P*-value = 0.0029). Homozygous *meRAD51C* had a greater reduction in *RAD51C* expression (Figure 7C) and a higher HRD score (39 versus 2–17; Figure 7B–D), when compared to the samples with heterozygous *meRAD51C*. Although the HRD score for the homozygous *meRAD51C* sample fell below the conventional threshold of 42 (Figure 7D) commonly used to predict HRD in BC and OV, the HRD score has not been validated for STAD (39). To determine the clinical consequence of *meRAD51C* in STAD cell lines, we assessed response to Olaparib (PARPi). SNU601 with homozygous *meRAD51C* displayed a greater sensitivity to Olaparib compared to the heterozygous *meRAD51C* cell-lines (Figure 7E). Furthermore, our results revealed that the conventional HRD score threshold did not align with Olaparib sensitivity in STAD; only one out of 14 unmethylated cell-lines with an HRD score greater than 42 displayed a higher sensitivity to Olaparib than SNU601 (Figure 7E), for which we did not find a genomic explanation.

## Discussion

While the impacts of me*BRCA1* and me*RAD51C* have been extensively studied in OV and BC (12,34,35,40,41), their implications in other cancer types remain largely unexplored. In this study, we characterised me*BRCA1* and me*RAD51C* and associated molecular and clinical features across multiple solid cancer types. We demonstrated variable prevalence of these two methylation events across cancer types, ranging from rare (e.g. *BRCA1* methylation in lung cancer) to highly prevalent (e.g. *RAD51C* methylation in STAD and *BRCA1* methylation in individuals of African ancestry with BC). Our findings suggest the two epigenetic alterations, *meBRCA1* and *meRAD51C*, stand as distinctive molecular events that vary across cancer type, dependent upon molecular subtype, with distinct methylation profiles, molecular consequence, and associated driver events.

*BRCA1* methylation was detected in several cancer types, albeit as a rare event. Only OV, BC and UCEC had a *BRCA1* methylation frequency of 1% or more, which was further enriched in specific molecular subgroups. In BC, me*BRCA1* was enriched in TNBC, as expected (9). This corresponded to an enrichment of both *meBRCA1* and *meRAD51C*, observed in >10% of individuals of African ancestry with BC, where the triple-negative subtype is more prevalent (42), with potentially important clinical implications, as it may contribute to the observation of a poorer prognosis in this context (42).

We also identified *BRCA1* methylation in UCEC, with all cases found in the p53-mutant copy number (CN) high subgroup, which is a subgroup correlated with unfavourable clinical outcomes (43). To our knowledge this is the first report of *BRCA1* methylation in endometrial carcinoma, with one previous report of *BRCA1* methylation in uterine leiomyosarcoma (44). We identified a consistent correlation between *BRCA1* methylation and genomic biomarkers for HRD across all cancer types with *BRCA1* methylation. The concurrent presence of *TP53* mutations was also observed, known to be a key element for the survival of cells with inactivated *BRCA1* function, as demonstrated by Xu *et al.* (45) and Na *et al.* (46). While occurrence of *BRCA1* methylation was relatively rare across all cancer types, its clinical significance and potential therapeutic implications warrant further investigation. In particular, given the rise in precision-personalised medicine and the successful N-of-1 treatment approach demonstrated in the I-PREDICT study (47), *BRCA1* methylation beyond OV and BC should be explored as a potential biomarker for PARPi sensitivity, particularly in the CN high endometrial subgroup, where we observed *BRCA1* methylation at >3% frequency. Furthermore, existing HRD genomic scarring biomarkers may not be appropriate for cancers with frequent genomic instability not caused by HRD, due to reduced specificity. As such, tumour-corrected promoter methylation testing of HRR genes may offer a more suitable direct readout of the HRD status.

*RAD51C* methylation was observed more broadly across cancer types, especially in those characterised by the distinctive CpG island methylator phenotype, CIMP. However, unlike for *BRCA1* methylation, the relationship between *RAD51C* methylation and the HRD biomarker, was less straightforward. Whilst the previously observed (34,35,48) association between *RAD51C* methylation and HRD was validated for OV and TNBC, it was absent in other cancer types. With the exception of OV and TNBC, *RAD51C* methylation was also generally detected at a low tumour level (<70%, suggestive of heterozygous or subclonal status) and without accompanying LOH or HRD scarring. Furthermore, in stomach cancer, *RAD51C* methylated cases were characterised by mutual exclusivity with *TP53* mutations, the opposite trend to *BRCA1* methylation across multiple cancer types. Altogether, these observations point to different roles for *BRCA1* and *RAD51C* methylation in cancer development, progression and therapeutic potential.

Promoter methylation of both *BRCA1* and *RAD51C* has previously been reported as a constitutional event, likely arising during early embryogenesis before germ layer formation (49). Additionally, constitutional methylation is more frequently detected in OV and BC cases compared with controls (50,51), and is enriched in early-onset OV (52) and BC (10,50). In our analysis, we observed younger age at diagnosis for patients with *BRCA1*-methylated OV in two independent cohorts, confirming previous reports (53,54). Collectively, these observations support the contribution of constitutional methylation of *BRCA1* and *RAD51C* to OV and TNBC development.

**Figure 8.**
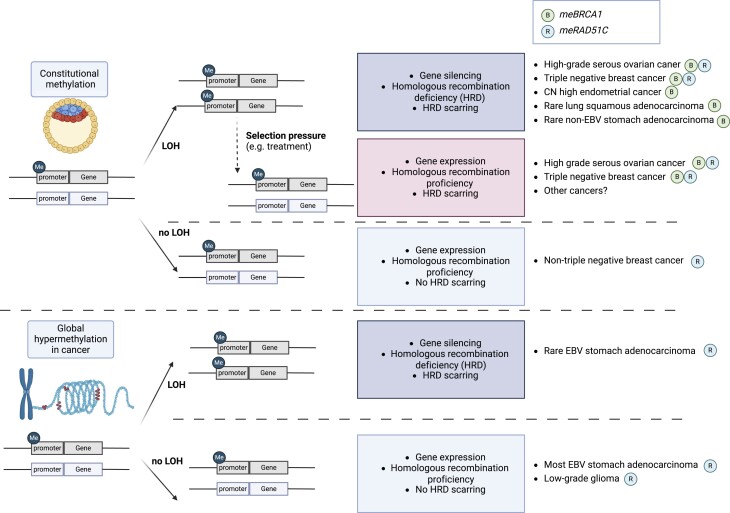
Proposed mechanism for variation in me*BRCA1* and me*RAD51C* methylation levels and impact on homologous recombination deficiency (HRD). The initial event of methylation may occur on one of the alleles. This could be a result of aberrant de novo methylation acquired as part of the epigenetic reprogramming during early embryogenesis (before germ layer formation), which has also been termed constitutional methylation. Alternatively, methylation could be established as part of the global promoter hypermethylation phenotype in cancer, for example in association with EBV infection. At this stage, the cell remains competent in homologous recombination repair. Subsequently, in some cases, a loss of heterozygosity ensues, leading to methylation in both alleles, resulting in an HRD phenotype and the accumulation of HRD genomic scarring. Finally, selection pressures, such as treatment exposure, can induce partial loss of methylation and consequently rescuing homologous recombination repair functionality, leading to PARP inhibitor resistance. Created with BioRender.com

In other non-CIMP-driven cancer types, including other subtypes of BC, it is not clear whether the observed tumour *BRCA1* or *RAD51C* methylation arose from constitutional methylation or from an early cancer-acquired event. We hypothesise that it is the former, but for other cancer types, *meRAD51C* may not result in a second LOH hit, as has been documented for OV and TNBC (13,14,41). Hence, cancer cells with heterozygous *RAD51C* promoter methylation likely retain HRR function, without the acquisition of the HRD phenotype. Taken together, we propose a model where the variation in *meBRCA1* and *meRAD51C* methylation levels and impact on HRD can be explained (Figure 8). On the other hand, in EBV-driven CIMP+ stomach cancer, *RAD51C* methylation is likely a consequence of the global CpG island methylation (55,56). Interestingly, *RAD51C* promoter was almost ubiquitously methylated in CIMP+ stomach cancer cases, but mostly at low tumour methylation level (likely heterozygous), suggesting that homozygous *RAD51C* methylation may offer a growth disadvantage in the stomach cancer development. Therapeutic relevance in this context is less clear, perhaps requiring combination, rather than single-agent PARPi therapy to induce tumour response.

Since HRD scarring was an infrequent event for *RAD51C* methylation beyond OV and TNBC, me*RAD51C* is unlikely to be relevant for most cancer types as a PARPi single-agent treatment biomarker. However, we did observe a subset of *RAD51C* methylated stomach cancer cases with LOH and evidence of HRD-associated scarring (albeit not at the HRD score threshold of 42). We also observed *in vitro* PARPi sensitivity for the homozygous *meRAD51C* cell-line compared with heterozygous *meRAD51C* cell-lines. Notably, the homozygous *RAD51C* methylated cell line was the only one established from a post-treatment sample, also containing LOH of *RAD51C*. Exploration of *RAD51C* methylation in the context of LOH and treatment-exposure would aid further characterisation with regard to its gene silencing effects, impact on HRD phenotype and therapeutic relevance across cancer types.

In addition to the initial establishment of *BRCA1* and *RAD51C* methylation, several studies have reported that complete or partial methylation loss, contributes to the development of resistance to platinum chemotherapy and PARPi (12,34). In OV, frequent methylation has been reported in cases treated with three or more cycles of chemotherapy (41). In this study, we identified a subset of primary OV and TNBC cases exhibiting low tumour level methylation in *BRCA1*. While we were unable to determine if all these samples were chemo-naïve, a recent report has documented two of 11 chemo-naïve TNBC cases as having low level *BRCA1* methylation (57). Considering the implication of *BRCA1* methylation loss for PARPi resistance (12,58), this observation raises crucial questions relevant for further exploration. Follow-up studies examining the frequency of low tumour *BRCA1* or *RAD51C* methylation in the chemo-naïve setting will be critical for understanding early treatment resistance in OV and BC.

The complexity of the tumour methylation level analysis, which is reliant on accurate tumour purity and copy number estimation, is a known limitation of *BRCA1* and *RAD51C* methylation characterisation studies. Moreover, the limited coverage of the *RAD51C* promoter region on methylation arrays and the presence of the heterogeneous *RAD51C* methylation pattern, further complicates assessments of methylation level. Additionally, refinement and validation of methods to assess HRD genomic scarring in non-OV/BC types is needed to gain a comprehensive understanding of the role of *BRCA1* and *RAD51C* methylation in tumourigenesis and drug resistance. Finally, future studies should consider population size and diversity, given the rare frequency of *BRCA1* and *RAD51C* methylation in some cancers, and differences in molecular subtype-enrichment across populations and ancestries (e.g. TNBC and CN high endometrial cancer in individuals of African ancestry (42,59) or EBV-associated stomach cancer frequencies across populations (60,61)).

In conclusion, this pan-cancer study broadens our understanding of *BRCA1* and *RAD51C* methylation and identifies important avenues for further exploration, especially due to significant therapeutic implications. As we navigate the complexity of cancer epigenetics, the interplay between DNA methylation, histone modifications and genomic events present new opportunities for translational discoveries to drive novel therapeutic interventions and enhance patient outcomes.

## Supplementary Material

zcae033_Supplemental_Files

## Data Availability

This project used publicly available dataset from TCGA GDC portal (https://portal.gdc.cancer.gov/) and cBioPortal (https://www.cbioportal.org/). ICGC methylation data is available from Gene Expression Omnibus (GEO) under the accession GSE6582. The whole genome and transcriptome sequencing data is available from European Genome-phenome Archive (EGA) repository under the accession code EGAD00001000877. DepMap mutation and drug sensitivity data is accessible from https://depmap.org. The raw genomic data is available on the Sequence Read Archive (SRA) under the accession number PRJNA523380. The source table containing beta values per probe per patient is available from https://doi.org/10.6084/m9.figshare.25249060.v1.

## Data availability

The R code (R version 4.0.2) used for analyses in this study is available from https://doi.org/10.6084/m9.figshare.25249060.v1.
