# Supplementary material for: High-level tumour methylation of BRCA1 and RAD51C is required for homologous recombination deficiency in solid cancers
Source: NAR Cancer. 2024 Jul 25;6(3):zcae033. doi: 10.1093/narcan/zcae033 (PMC11270467; doi:10.1093/narcan/zcae033)
Supplement: zcae033_Supplemental_Files [file zcae033_supplemental_files.zip › Xu_etal_Pan-cancer_methylation_supplementary methods_clean.docx]

**Supplementary Methods:**

**High-level tumour methylation of *BRCA1* and *RAD51C* is required for homologous recombination deficiency in solid cancers**

Lijun Xu^1,2^, Brett Liddell^1,2^, Ksenija Nesic^3^, Franziska Geissler^3^, Matthew J. Wakefield^3, 4^, Clare L. Scott^3, 4^, Lauren M. Ashwood^1,2^, Nicola Waddell^1,2,†^ and Olga Kondrashova^1,2,3,†^

^1^Cancer Research Program, QIMR Berghofer Medical Research Institute, Brisbane, QLD, Australia

^2^The University of Queensland, Brisbane, QLD, Australia

^3^The Walter and Eliza Hall Institute of Medical Research, Parkville, VIC, Australia

^4^Department of Obstetrics and Gynaecology, University of Melbourne, Parkville, VIC, Australia.

^†^These authors jointly supervised this work.

Corresponding author:

Olga Kondrashova,

300 Herston Road, Herston,

4006, QLD, Australia

[olga.kondrashova@qimrberghofer.edu.au](mailto:olga.kondrashova@qimrberghofer.edu.au)

**Supplementary Methods**

*Methylation Pre-processing*

The raw HM450K readouts were background corrected, normalised to internal controls and quality controlled at the probe and sample level using R package ChAMP (v2.16.2)(1). Additional filtration was applied to remove SNP related probes. For patient with multiple primary samples available, a single sample per case was selected based on the best QC metric (the lowest fraction of failed probes) for downstream analysis after promoter methylation status assessed.

For DepMap, reduced representation bisulfite sequencing data for 33 STAD cell-lines was obtained Sequence Read Archive (SRA) under the accession number PRJNA523380 (accessed in Apr 2023). The trimmed reads were aligned against reference human genome build hg19 using Bismark (v0.19.1) and methylation data for CpG sites was selected for analysis using the methylKit package (v1.16.1). Only sites with a minimum coverage of 5 and a minimum Phred score of 20 were retained.

*Selection of Promoter-Associated Probes*

Promoter region for *BRCA1, BRCA2, RAD51B, RAD51C, RAD51D, PALB2*, *XRCC3* and *BRIP1* were obtained from EPDnew (v006)(2) for hg38 assembly, based on GENCODE version 28. Specifically for *BRCA1*, only promoters proximate to transcription start site and previously associated with *BRCA1* expression(3) were included. These regions were then extended by 200 base pairs upstream and downstream. In cases where CpG islands overlapped with the promoter region and continued beyond the 200-base pair boundary, the considered region was further extended to include CpG island. Probes within the considered regions were examined, and their characteristics were assessed based on the median and variability observed across all tumor and normal samples included in this study (Table S1-1). Probes with median methylation level ≤0.2 and an interquartile range (IQR) ≤0.2 were retained. If the boundary (first or last) probes within the considered region met these criteria, adjacent CpG probes were also evaluated. If the adjacent probes also met the selection criteria, the region was further extended until probes failed to meet the specified thresholds. The detailed probe set for each gene is provided in Table S1.

*Promoter Methylation*

Ensuring the probe set accurately reflects aberrant methylation levels in tumour samples without capturing high methylation background in normal tissue, we assessed the median methylation levels for each of the eight HRR genes across different cancer types (Figure S1). We calculated the median methylation level of specific gene probes, ensuring the highest median methylation did not surpass 0.25 in normal tissue. This step aimed to minimize background methylation noise and enhance our ability to detect promoter methylation. In breast cancer (BC), *BRIP1* and *RAD51B* were not evaluated for promoter methylation because their highest median methylation levels in normal breast samples exceeded 0.25 (Figure S1; Table S1-S2).

Promoter methylation in the provided primary tumour samples was determined by assessing at least 60% of selected probes for each gene (rounded to the closest integer), with a methylation estimation of ≥0.25. The threshold for the number of probes required to indicate methylation for each gene is as follows:

*BRCA1* (13 out of 21 probes), *BRCA2* (eight out of 13 probes), *BRIP1* (five out of nine probes), *PALB2* (10 out of 16 probes), *RAD51B* (three out of five probes), *RAD51C* (four out of seven probes), *RAD51D* (seven out of 12 probes), and *XRCC3* (six out of 10 probes).

*Cancer Subtype and Patient information*

Histological subtype for all TCGA samples and molecular subtypes for STAD, LGG, UCEC were retrieved from cBioPortal (accessed in May 2023). Molecular subtype for TCGA BC dataset was sourced from Thennavan *et al*. (2021)(4), while the molecular subtype for ICGC OV dataset was derived from Patch *et al*. (2015)(5).

Patient information including stage at diagnosis, age at diagnosis, sex was accessed through cBioPortal (accessed in May 2023).

Inferred ancestral information from SNP array was obtained from Carrot-Zhang’s study(6).

*Survival Analysis*

The Kaplan-Meier survival analysis for the overall survival (OS) was performed using the curated clinical data from Liu J et al.(7) (TCGA) and Patch *et al*.(5) (ICGC). The statistical testing and plotting were done with package survminer (version 0.4.9). The curve comparison was performed with the log-rank test.

*CIMP Classification*

We used CIMP classification from Yates *et al*. (2022)(8) for TCGA datasets, including STAD, LGG, BC and UCEC. For ICGC OV and TCGA TGCT datasets, the classification was not available from the publication. Therefore, we adopted the same approach as Yates *et al*. We filtered CpG probes that were non-CpG island located, sex-linked, non-variable (determined through k-means clustering based on standard deviation of the beta values), and age-related (identified by multiple linear regression between beta values and age, with a significance threshold of p < 0.05). For OV, two out of 81 samples were removed due to poor purity and clustering was performed across a set of 96,840 probes. For TGCT, one out of 150 samples was removed as it failed QC. The subsequent clustering was performed on 143,659 probes.

For DepMap, we filtered for autosomal sites within CpG islands, shores and shelves (+/-4kb) that showed variation in their methylation (SD≥0.3). To estimate CIMP status, we used hierarchical clustering with complete linkage from R packages stats (v4.2.0) and ComplexHeatmap (v2.14.0).

*Mutation Calling, Prevalence and Variant Annotation*

For TCGA, we obtained pre-processed somatic base changes and small insertion/deletion (indels) variants from the Multi-Center Mutation Calling in Multiple Cancers project (MC3) (9). For ICGC, the method used to detect substitution and indels is reported in Patch *et al*.(5).

To obtain non-silent mutations for the assessment of prevalence and tumour mutation burden, filtration was performed on the somatic mutation to remove 5 prime UTR variants, 3 prime UTR variants, synonymous variants, or variants in introns, 3 prime flank or 5 prime flank. For the prevalence assessment, we focused on 1,097 tumour driver genes that have been curated by OncoKB^TM^ (list obtained in April 2023).

Information about clinically significant HRR gene mutations (including somatic and germline mutation in *BRCA1, BRCA2, CDK12, RAD51C, PALB2, BRIP1* and *RAD51D*) in ICGC ovarian cohort was obtained from Patch *et al*.(5).

The variant effect was predicted using Ensembl Variant Effect Predictor(10) (v102) and annotations from the ClinVar database(11) (accessed in Oct 2022). Clinically significant variants were selected according to the review status (variants with interpretation and assertion criteria and without conflicting interpretation), clinical significance (pathogenic or likely pathogenic) and potential impact on transcription (variants cause stop gain or frameshift and occur at a location other than the last exon).

For DepMap, ClinVar annotation of the somatic mutation was performed using the same methods as TCGA.

*Gene Silencing Effect*

The pre-processed gene counts for TCGA mRNA sequencing were obtained from the GDC (<https://docs.gdc.cancer.gov/Data/Bioinformatics_Pipelines/Expression_mRNA_Pipeline/>). The ICGC mRNA sequencing data was processed using the method described in Patch *et al*.(5).

For patients with multiple primary sample available, the samples with the highest total gene count were selected for downstream analyses. The transcripts were filtered for protein coding gene with minimal expression level at 10 count-per-million (CPM) or higher in at least in 3 samples. The library size was recalculated and normalised with the factor of 1, followed by transformation into log2 CPM for downstream analysis.

For DepMap, mRNA sequencing data was processed using the method as described by Patch *et al*.(5). The library size was recalculated and normalised with the factor of 1, followed by transformation into log2 CPM for downstream analysis.

*Gene-Level and Allele-Specific Copy Number Alterations*

For TCGA, we utilised pre-processed allele-specific copy number segments obtained through ASCAT3, as conducted by the Vanloo lab(12). In cases where ASCAT3 data was unavailable for three samples (TCGA-HU-A4H0, TCGA-VQ-A923, TCGA-AO-A0JB), we employed results obtained from ASCAT2. For ICGC, we used copy number segment information from whole genome sequencing (WGS) data using ascatNGS(13)(v4.0.1).

For DepMap, the pre-processed copy number and LOH information was obtained from the CCLE ABSOLUTE (CCLE_ABSOLUTE_COMBINED_20181227, 2019 release). HRD score was calculated using scarHRD package(14) (v0.1.1).

*HRD Score and Signature analysis*

For TCGA, we access the pre-calculated whole exome sequencing derived HRD score from Knijnenburg *et al*.(15). For ICGC, the HRD score were calculated using scarHRD package(14) (v0.1.1). The estimation was dominantly performed on the allele-specific copy number derived from whole genome sequencing (WGS) data using ascatNGS(13)(v4.0.1). For the two samples with no WGS data available for the primary tumour, copy number derived from SNP array by GAP(16) was used instead.

The contribution of the samples’ single nucleotide variants (SNV) to the 30 known COSMIC v2 signatures was estimated using deconstructSig(17) (v1.8.0). Somatic SNVs for TCGA and ICGC dataset was obtained as described in mutation calling.

*Tumour Purity*

For TCGA, we used pre-processed purity estimation(18), using the ABSOLUTE method(19) and copy number variations. For ICGC, we used the purity estimation derived from WGS data using ascatNGS(13) (v4.0.1).

References

1. Zhou, W., Laird, P.W. and Shen, H. (2017) Comprehensive characterization, annotation and innovative use of Infinium DNA methylation BeadChip probes. *Nucleic Acids Res*, **45**, e22.

2. Dreos, R., Ambrosini, G., Périer, R.C. and Bucher, P. (2015) The Eukaryotic Promoter Database: expansion of EPDnew and new promoter analysis tools. *Nucleic Acids Research*, **43**, D92-D96.

3. DiNardo, D.N.M., Butcher, D.T., Robinson, D.P., Archer, T.K. and Rodenhiser, D.I. (2001) Functional analysis of CpG methylation in the BRCA1 promoter region. *Oncogene*, **20**, 5331-5340.

4. Thennavan, A., Beca, F., Xia, Y., Recio, S.G., Allison, K., Collins, L.C., Tse, G.M., Chen, Y.Y., Schnitt, S.J., Hoadley, K.A. *et al.* (2021) Molecular analysis of TCGA breast cancer histologic types. *Cell Genom*, **1**.

5. Patch, A.M., Christie, E.L., Etemadmoghadam, D., Garsed, D.W., George, J., Fereday, S., Nones, K., Cowin, P., Alsop, K., Bailey, P.J. *et al.* (2015) Whole-genome characterization of chemoresistant ovarian cancer. *Nature*, **521**, 489-494.

6. Carrot-Zhang, J., Chambwe, N., Damrauer, J.S., Knijnenburg, T.A., Robertson, A.G., Yau, C., Zhou, W., Berger, A.C., Huang, K.-l., Newberg, J.Y. *et al.* (2020) Comprehensive Analysis of Genetic Ancestry and Its Molecular Correlates in Cancer. *Cancer Cell*, **37**, 639-654.e636.

7. Liu, J., Lichtenberg, T., Hoadley, K.A., Poisson, L.M., Lazar, A.J., Cherniack, A.D., Kovatich, A.J., Benz, C.C., Levine, D.A., Lee, A.V. *et al.* (2018) An Integrated TCGA Pan-Cancer Clinical Data Resource to Drive High-Quality Survival Outcome Analytics. *Cell*, **173**, 400-416 e411.

8. Yates, J. and Boeva, V. (2022) Deciphering the etiology and role in oncogenic transformation of the CpG island methylator phenotype: a pan-cancer analysis. *Briefings in Bioinformatics*, **23**, bbab610.

9. Ellrott, K., Bailey, M.H., Saksena, G., Covington, K.R., Kandoth, C., Stewart, C., Hess, J., Ma, S., Chiotti, K.E., McLellan, M. *et al.* (2018) Scalable Open Science Approach for Mutation Calling of Tumor Exomes Using Multiple Genomic Pipelines. *Cell Syst*, **6**, 271-281.e277.

10. McLaren, W., Gil, L., Hunt, S.E., Riat, H.S., Ritchie, G.R., Thormann, A., Flicek, P. and Cunningham, F. (2016) The Ensembl Variant Effect Predictor. *Genome Biol*, **17**, 122.

11. Landrum, M.J., Lee, J.M., Benson, M., Brown, G.R., Chao, C., Chitipiralla, S., Gu, B., Hart, J., Hoffman, D., Jang, W. *et al.* (2018) ClinVar: improving access to variant interpretations and supporting evidence. *Nucleic Acids Research*, **46**, D1062-D1067.

12. Ross, E.M., Haase, K., Van Loo, P. and Markowetz, F. (2021) Allele-specific multi-sample copy number segmentation in ASCAT. *Bioinformatics*, **37**, 1909-1911.

13. Raine, K.M., Van Loo, P., Wedge, D.C., Jones, D., Menzies, A., Butler, A.P., Teague, J.W., Tarpey, P., Nik-Zainal, S. and Campbell, P.J. (2016) ascatNgs: Identifying Somatically Acquired Copy-Number Alterations from Whole-Genome Sequencing Data. *Curr Protoc Bioinformatics*, **56**, 15 19 11-15 19 17.

14. Sztupinszki, Z., Diossy, M., Krzystanek, M., Reiniger, L., Csabai, I., Favero, F., Birkbak, N.J., Eklund, A.C., Syed, A. and Szallasi, Z. (2018) Migrating the SNP array-based homologous recombination deficiency measures to next generation sequencing data of breast cancer. *NPJ Breast Cancer*, **4**, 16.

15. Knijnenburg, T.A., Wang, L., Zimmermann, M.T., Chambwe, N., Gao, G.F., Cherniack, A.D., Fan, H., Shen, H., Way, G.P., Greene, C.S. *et al.* (2018) Genomic and Molecular Landscape of DNA Damage Repair Deficiency across The Cancer Genome Atlas. *Cell Rep*, **23**, 239-254 e236.

16. Popova, T., Manie, E., Stoppa-Lyonnet, D., Rigaill, G., Barillot, E. and Stern, M.H. (2009) Genome Alteration Print (GAP): a tool to visualize and mine complex cancer genomic profiles obtained by SNP arrays. *Genome Biol*, **10**, R128.

17. Rosenthal, R., McGranahan, N., Herrero, J., Taylor, B.S. and Swanton, C. (2016) DeconstructSigs: delineating mutational processes in single tumors distinguishes DNA repair deficiencies and patterns of carcinoma evolution. *Genome Biol*, **17**, 31.

18. Hoadley, K.A., Yau, C., Hinoue, T., Wolf, D.M., Lazar, A.J., Drill, E., Shen, R., Taylor, A.M., Cherniack, A.D., Thorsson, V. *et al.* (2018) Cell-of-Origin Patterns Dominate the Molecular Classification of 10,000 Tumors from 33 Types of Cancer. *Cell*, **173**, 291-304 e296.

19. Carter, S.L., Cibulskis, K., Helman, E., McKenna, A., Shen, H., Zack, T., Laird, P.W., Onofrio, R.C., Winckler, W., Weir, B.A. *et al.* (2012) Absolute quantification of somatic DNA alterations in human cancer. *Nat Biotechnol*, **30**, 413-421.

20. Cadieux, E.L., Tanić, M., Wilson, G.A., Baker, T., Dietzen, M., Dhami, P., Vaikkinen, H., Watkins, T.B.K., Kanu, N., Veeriah, S. *et al.* (2020) Copy number-aware deconvolution of tumor-normal DNA methylation profiles. *bioRxiv*, 2020.2011.2003.366252.
