# Supplementary material for: High-level tumour methylation of BRCA1 and RAD51C is required for homologous recombination deficiency in solid cancers
Source: NAR Cancer. 2024 Jul 25;6(3):zcae033. doi: 10.1093/narcan/zcae033 (PMC11270467; doi:10.1093/narcan/zcae033)
Supplement: zcae033_Supplemental_Files [file zcae033_supplemental_files.zip › Xu_etal_Pan-cancer_methylation_supp_Figures.pdf]

High-level tumour methylation of *BRCA1* and *RAD51C* is required for homologous recombination deficiency in solid cancers

Supplementary Figures

Lijun Xu[1,2], Brett Liddell[1,2], Ksenija Nesic[3], Franziska Geissler[3], Lauren M. Ashwood[1,2], Matthew J. Wakefield[3, 4], Clare L. Scott[3, 4], Nicola Waddell[1,2,†] and Olga Kondrashova[1,2,3,†]

[1]Cancer Research Program, QIMR Berghofer Medical Research Institute, Brisbane, QLD, Australia

[2]The University of Queensland, Brisbane, QLD, Australia

[3]The Walter and Eliza Hall Institute of Medical Research, Parkville, VIC, Australia

[4]Department of Obstetrics and Gynaecology, University of Melbourne, Parkville, VIC, Australia.

[†]These authors jointly supervised this work.

Corresponding author:

Olga Kondrashova,

300 Herston Road, Herston,
4006, QLD, Australia

olga.kondrashova@qimrberghofer.edu.au

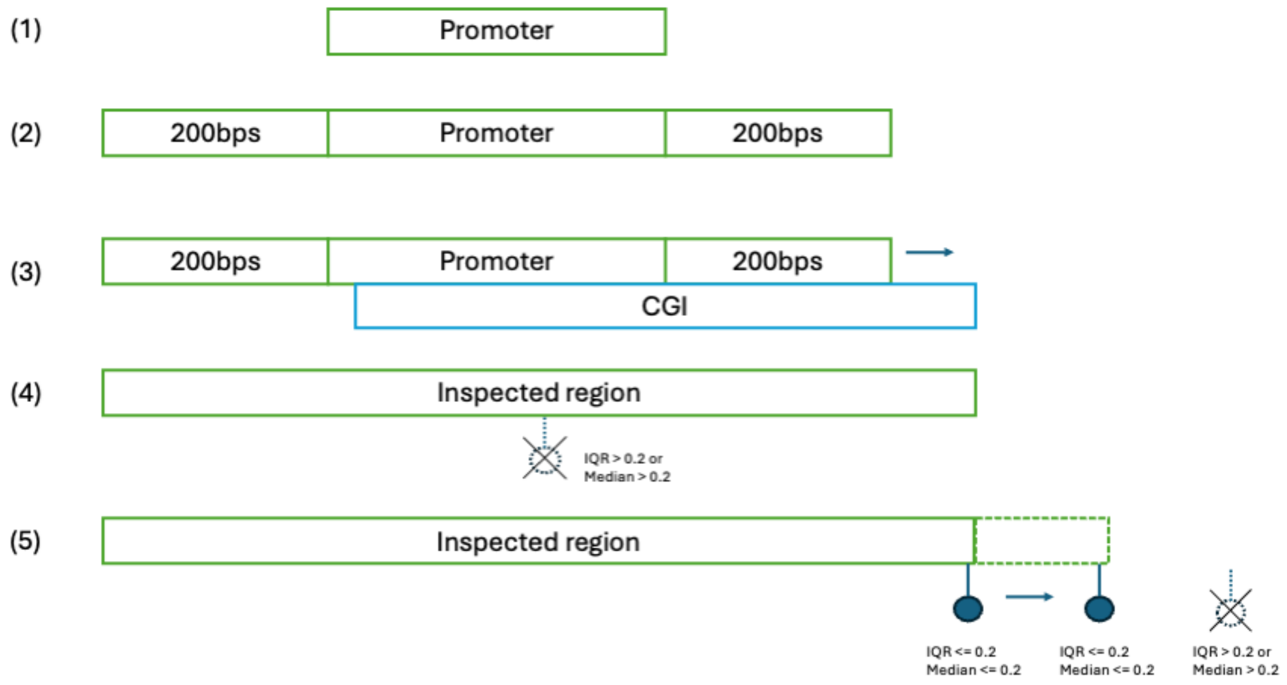

(1)
Promoter
(2)
200bps
Promoter
200bps
(3)
200bps
Promoter
200bps
CGI
(4)
Inspected region
IQR > 0.2 or
Median > 0.2
(5)
Inspected region
IQR <= 0.2
Median <= 0.2
IQR <= 0.2
Median <= 0.2
IQR > 0.2 or
Median > 0.2

Figure S1. Schematic for the selection of promoter associated probes. 1. Selection of Promoter Region: The initial step involved identifying the promoter region using the EPDnew v006 promoter annotation for the hg38 assembly, accessible at https://epd.expasy.org/epd/. For *BRCA1*, we only included the promoter regions (BRCA1_1, BRCA1_3 and BRCA1_4) that have been previously shown to be associated with *BRCA1* expression (DiNardo et al. 2001, Oncogene, 20, pp. 5331-5340). 2. Extension of Promoter Region: The identified promoter region was extended by 200 base pairs both upstream and downstream. 3. Consideration of CpG Islands (CGIs): If any CpG island overlapped with the extended promoter region and extended beyond the 200-base pair boundary, the region was further extended accordingly. 4. Probe Evaluation and Selection: Probes within the extended region were examined, and their characteristics were assessed based on the median methylation and variability observed across tumour and normal samples included in this study. Probes exhibiting a median methylation level less than or equal to 0.2 and an interquartile range (IQR) less than or equal to 0.2 were retained. Detailed information for each selected probe can be found in Table S1. 5. Expansion of Region Based on Adjacent Probes: If the terminal probes within the extended region met the criteria, adjacent CpG probes were evaluated. If these adjacent probes also met the selection criteria, the region was further extended until probes failed to meet the specified thresholds.

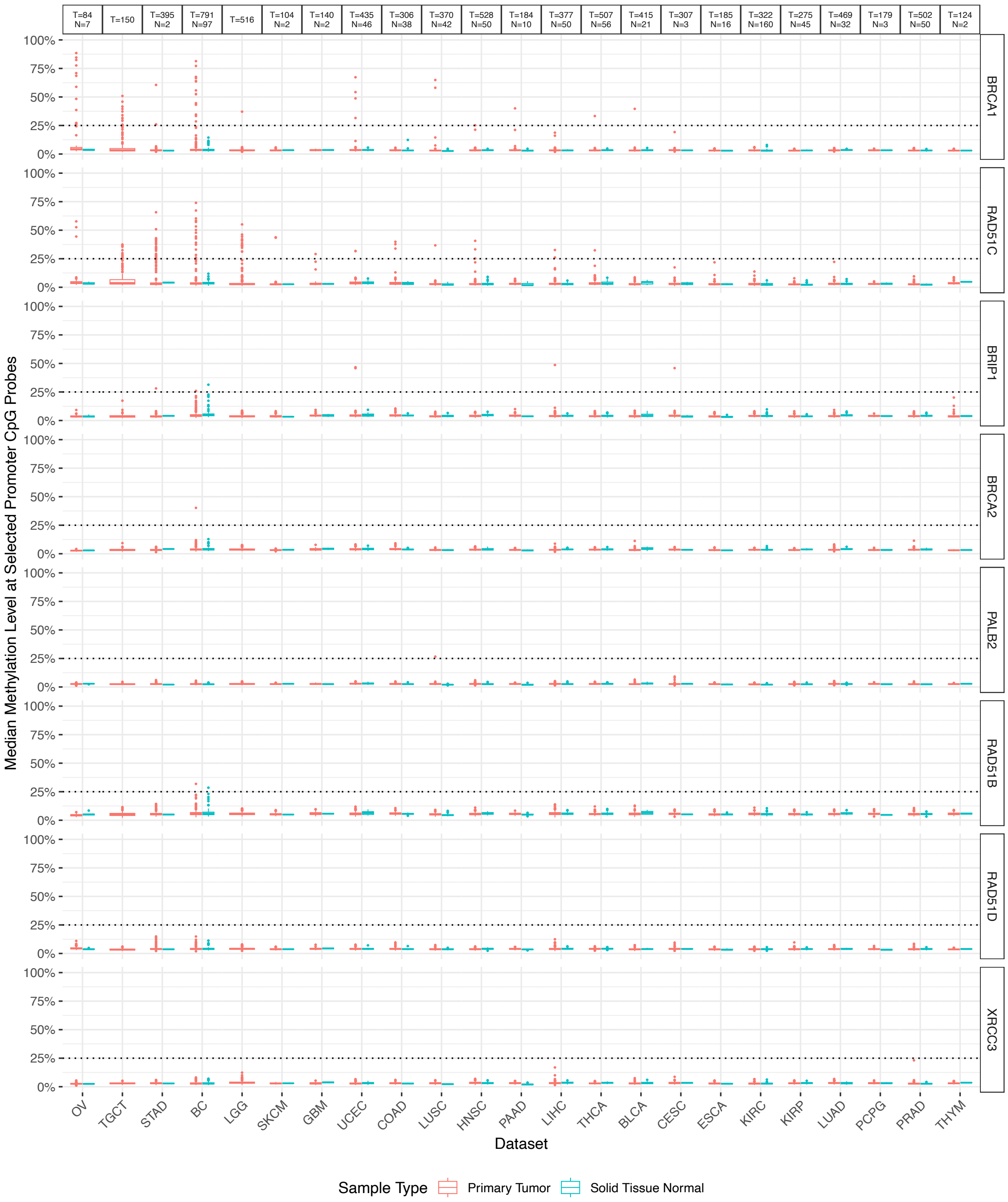

T=84 N=7
T=150
T=395 N=2
T=791 N=97
T=516
T=104 N=2
T=140 N=2
T=435 N=46
T=306 N=38
T=370 N=42
T=528 N=50
T=184 N=10
T=377 N=50
T=507 N=56
T=415 N=21
T=307 N=3
T=185 N=16
T=322 N=160
T=275 N=45
T=469 N=32
T=179 N=3
T=502 N=50
T=124 N=2
BRCA1
RAD51C
BRIP1
BRCA2
PALB2
RAD51B
RAD51D
XRCC3
100%
75%
50%
25%
0%
Median Methylation Level at Selected Promoter CpG Probes
OV
TGCT
STAD
BC
LGG
SKCM
GBM
UCEC
COAD
LUSC
HNSC
PAAD
LIHC
THCA
BLCA
CESC
ESCA
KIRC
KIRP
LUAD
PCPG
PRAD
THYM
Dataset
Sample Type
Primary Tumor
Solid Tissue Normal

Figure S2. Distribution for Average methylation level of selected probes covering promoter region for HRR genes (*BRCA1*, *RAD51C*, *BRIP1*, *BRCA2*, *PALB2*, *RAD51B*, *RAD51D* and *XRCC3*) within a sample. The box plots illustrate the distribution of average beta values from a subset of probes that cover and extend from the promoter region and promoter-overlapping CpG island (CGI) in both tumour samples (red) and normal tissue (blue). Each boxplot includes the median (center bar) and interquartile range (IQR), with the whiskers extending to the maximum and minimum values, but no further than 1.5 times the IQR. Outliers, defined as values beyond 1.5 times the IQR, are represented by dots above or below the box. The sample size for each cancer type is indicated for tumour (T) and normal (N) tissue samples in the top panel.

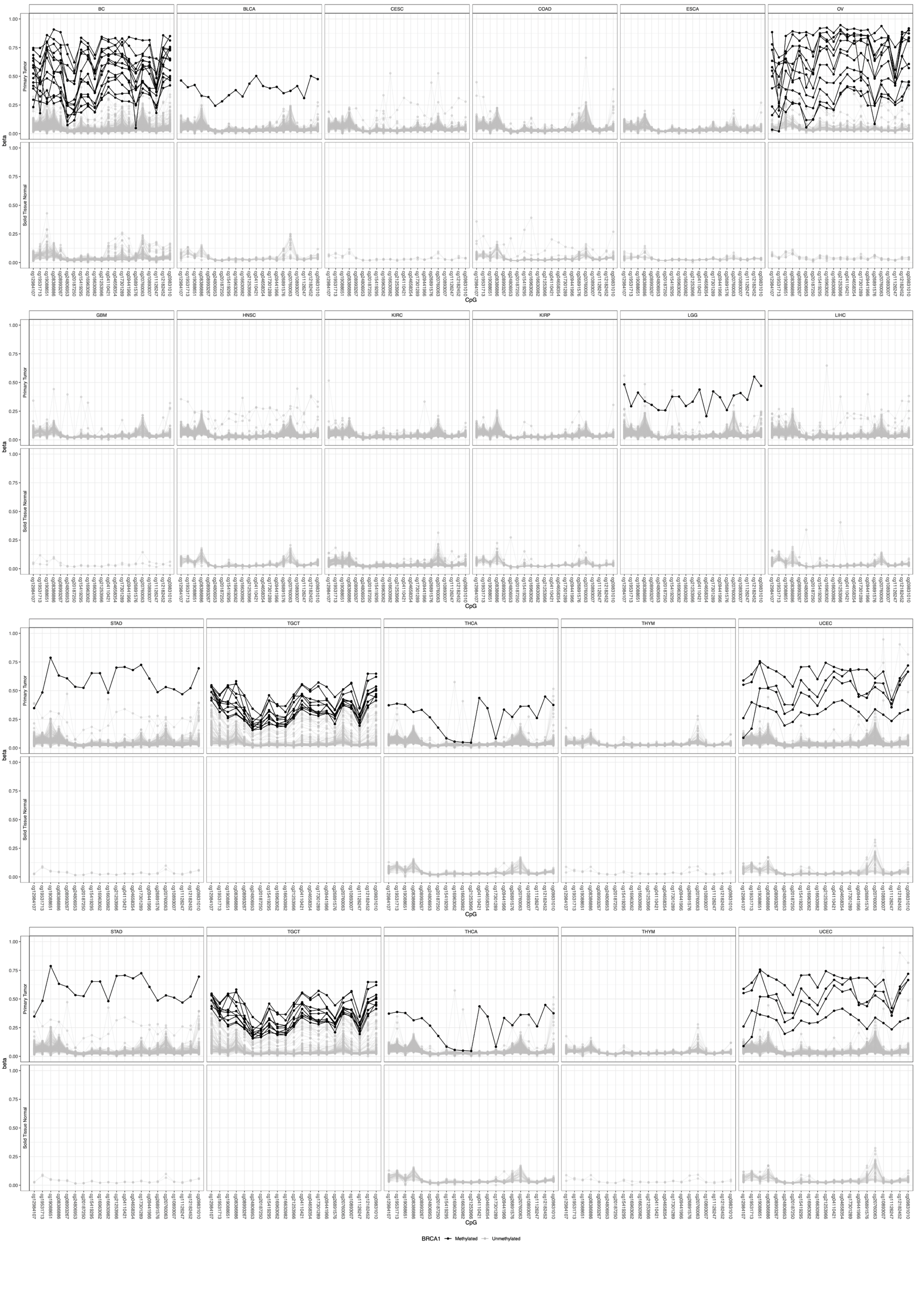
BC
BLCA
CESC
COAD
ESCA
OV
GBM
HNSC
KIRC
KIRP
LGG
LIHC
STAD
TGCT
THCA
THYM
UCEC
Primary Tumor
Solid Tissue Normal
beta
CpG
BRCA1
Methylated
Unmethylated

Figure S3. Methylation values at *BRCA1* promoter CpG probes. The plots depicting the methylation profiles of individual primary tumours and the normal samples for each analysed cancer type are shown. Individual set of dots connected by a line represent the estimated beta values from each case at the corresponding CpG (grey: predicted unmethylated, black: predicted promoter methylated). CpG probes (x-axis) are arranged in order of genomic location, however distances are not scaled by genomic coordinates. Only CpG probes selected for analysis are shown.

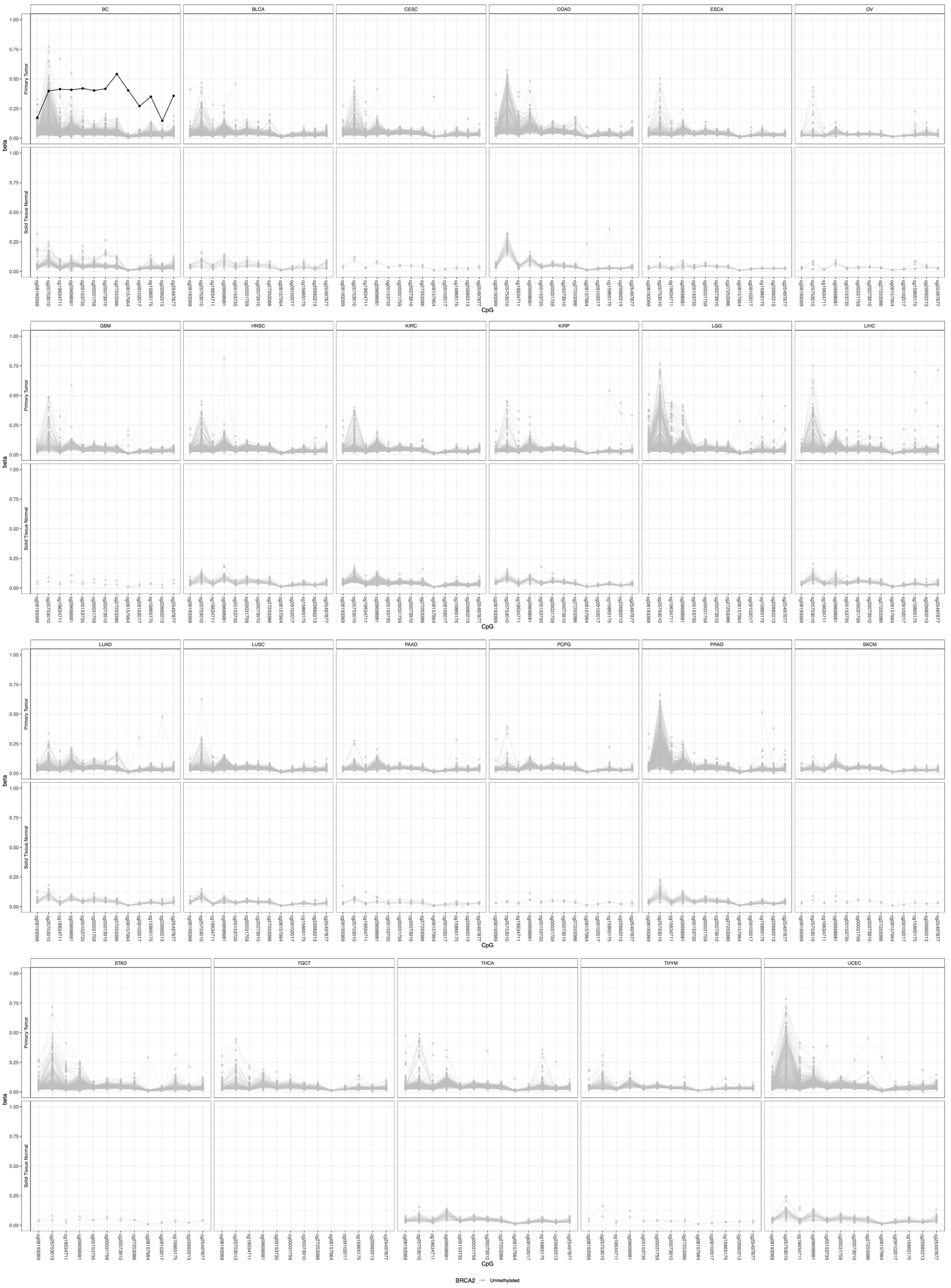
BC
BLCA
CESC
COAD
ESCA
OV
GBM
HNSC
KIRC
KIRP
LGG
LIHC
LUAD
LUSC
PAAD
PCPG
PRAD
SKCM
STAD
TGCT
THCA
THYM
UCEC
Primary Tumor
Solid Tissue Normal
beta
1.00
0.75
0.50
0.25
0.00
cg08193369
cg25753010
cg19634711
cg09698881
cg05153735
cg00031759
cg20078910
cg27253386
cg08157964
cg09102017
cg15865175
cg22682213
cg25497877
CpG
BRCA2
Unmethylated

Figure S4. Methylation values at *BRCA2* promoter CpG probes. The plots depicting the methylation profiles of individual primary tumours and the normal samples for each analysed cancer type are shown. Individual set of dots connected by a line represent the estimated beta values from each case at the corresponding CpG (grey: predicted unmethylated, black: predicted promoter methylated). CpG probes (x-axis) are arranged in order of genomic location, however distances are not scaled by genomic coordinates. Only CpG probes selected for analysis are shown.

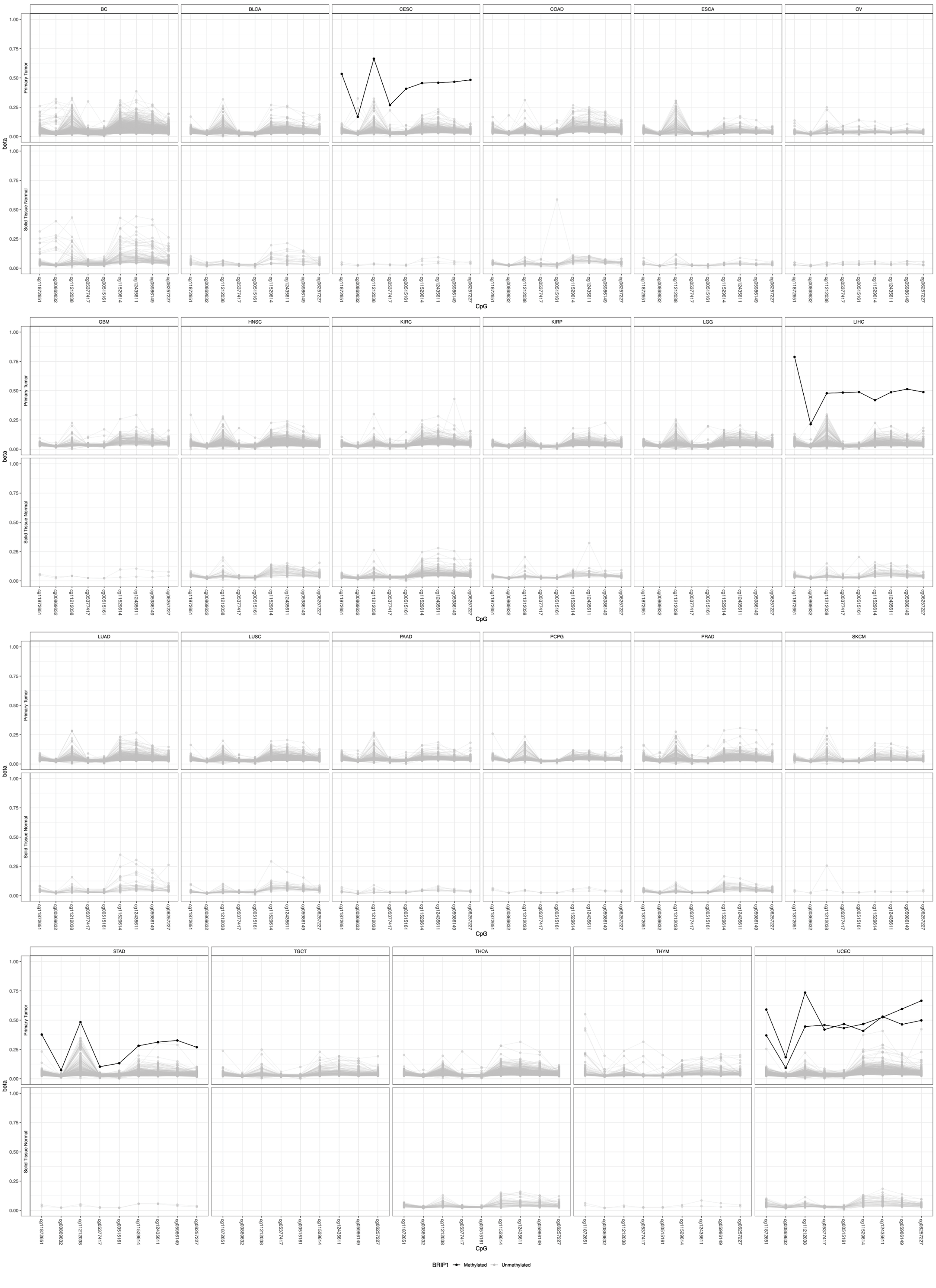
BC
BLCA
CESC
COAD
ESCA
OV
GBM
HNSC
KIRC
KIRP
LGG
LIHC
LUAD
LUSC
PAAD
PCPG
PRAD
SKCM
STAD
TGCT
THCA
THYM
UCEC
Primary Tumor
Solid Tissue Normal
beta
1.00
0.75
0.50
0.25
0.00
cg11872651
cg00869632
cg11212038
cg05377417
cg00515161
cg11529614
cg12435611
cg05986149
cg06257227
CpG
BRIP1
Methylated
Unmethylated

Figure S5. Methylation values at *BRIP1* promoter CpG probes. The plots depicting the methylation profiles of individual primary tumours and the normal samples for each analysed cancer type are shown. Individual set of dots connected by a line represent the estimated beta values from each case at the corresponding CpG (grey: predicted unmethylated, black: predicted promoter methylated). CpG probes (x-axis) are arranged in order of genomic location, however distances are not scaled by genomic coordinates. Only CpG probes selected for analysis are shown.

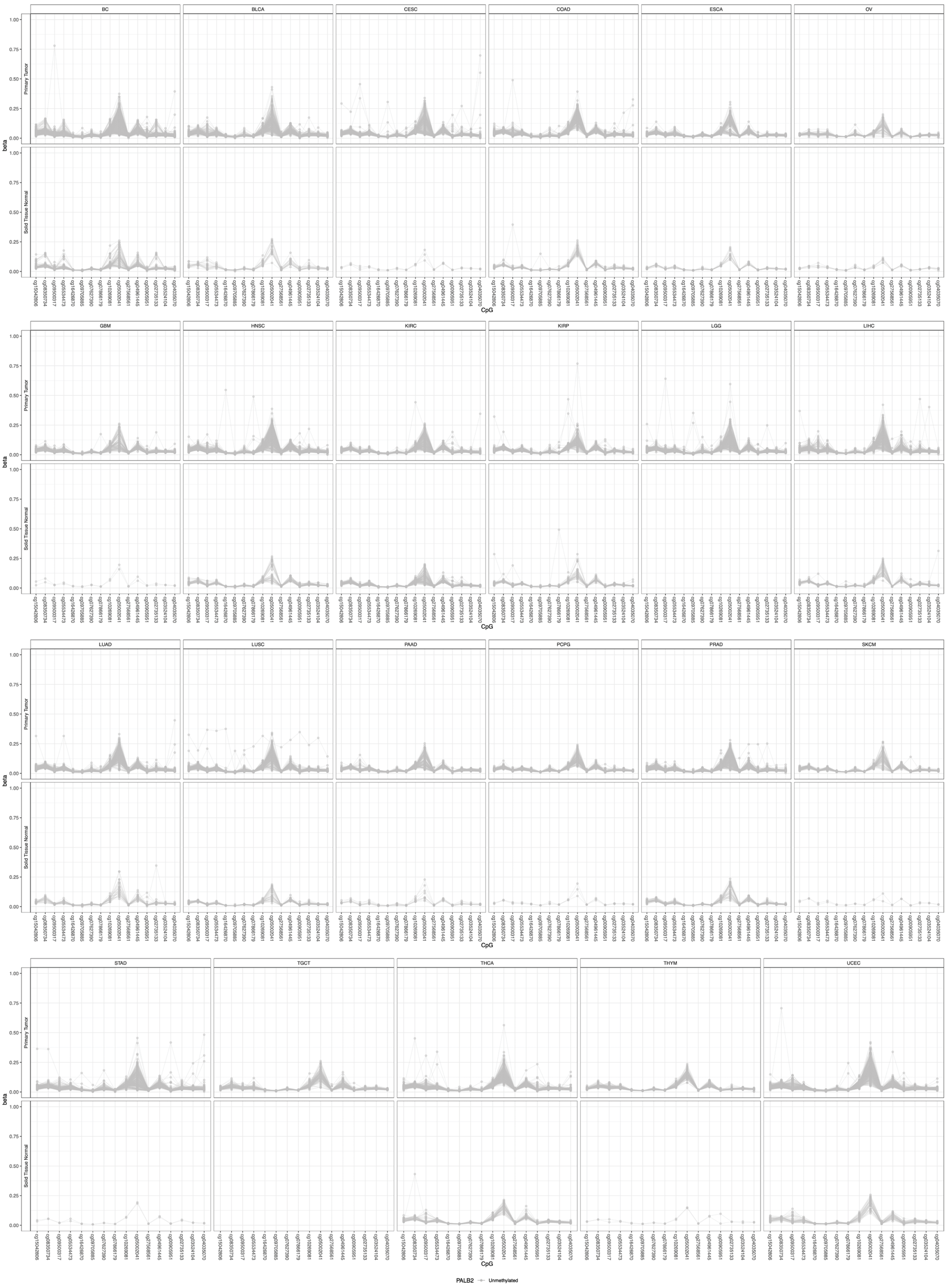
BC
BLCA
CESC
COAD
ESCA
OV
GBM
HNSC
KIRC
KIRP
LGG
LIHC
LUAD
LUSC
PAAD
PCPG
PRAD
SKCM
STAD
TGCT
THCA
THYM
UCEC
Primary Tumor
Solid Tissue Normal
beta
CpG
0.00
0.25
0.50
0.75
1.00
cg15042806
cg08350734
cg09500017
cg05534473
cg16428670
cg09705885
cg07627390
cg07866179
cg10269081
cg05002041
cg27568561
cg04961445
cg00005951
cg02735133
cg03524104
cg04035070
PALB2
Unmethylated

Figure S6. Methylation values at *PALB2* promoter CpG probes. The plots depicting the methylation profiles of individual primary tumours and the normal samples for each analysed cancer type are shown. Individual set of dots connected by a line represent the estimated beta values from each case at the corresponding CpG (grey: predicted unmethylated, black: predicted promoter methylated). CpG probes (x-axis) are arranged in order of genomic location, however distances are not scaled by genomic coordinates. Only CpG probes selected for analysis are shown.

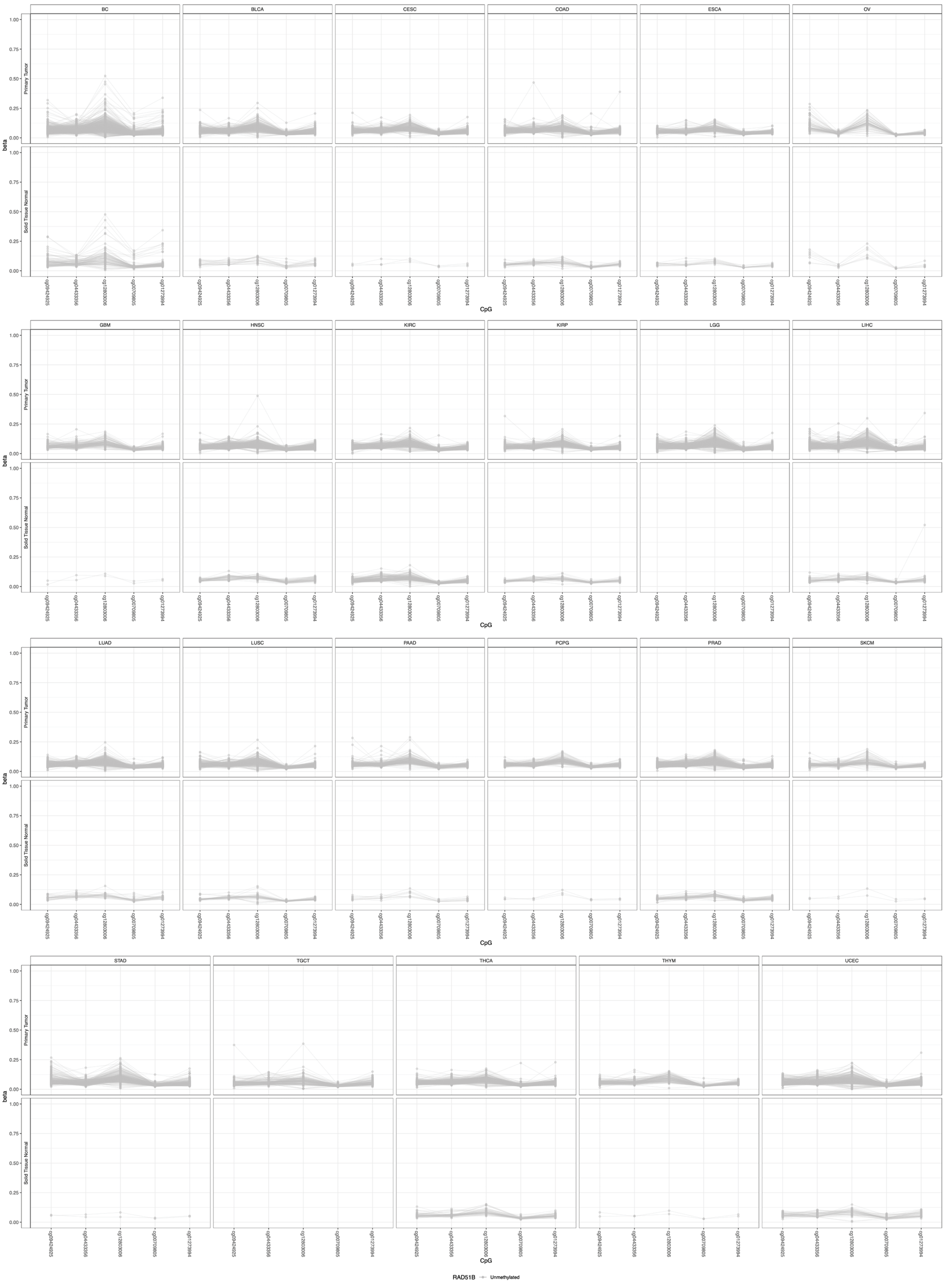
BC
BLCA
CESC
COAD
ESCA
OV
GBM
HNSC
KIRC
KIRP
LGG
LIHC
LUAD
LUSC
PAAD
PCPG
PRAD
SKCM
STAD
TGCT
THCA
THYM
UCEC
Primary Tumor
Solid Tissue Normal
beta
CpG
cg09424925
cg04433356
cg12803006
cg00709805
cg01273994
RAD51B
Unmethylated

Figure S7. Methylation values at *RAD51B* promoter CpG probes. The plots depicting the methylation profiles of individual primary tumours and the normal samples for each analysed cancer type are shown. Individual set of dots connected by a line represent the estimated beta values from each case at the corresponding CpG (grey: predicted unmethylated, black: predicted promoter methylated). CpG probes (x-axis) are arranged in order of genomic location, however distances are not scaled by genomic coordinates. Only CpG probes selected for analysis are shown.

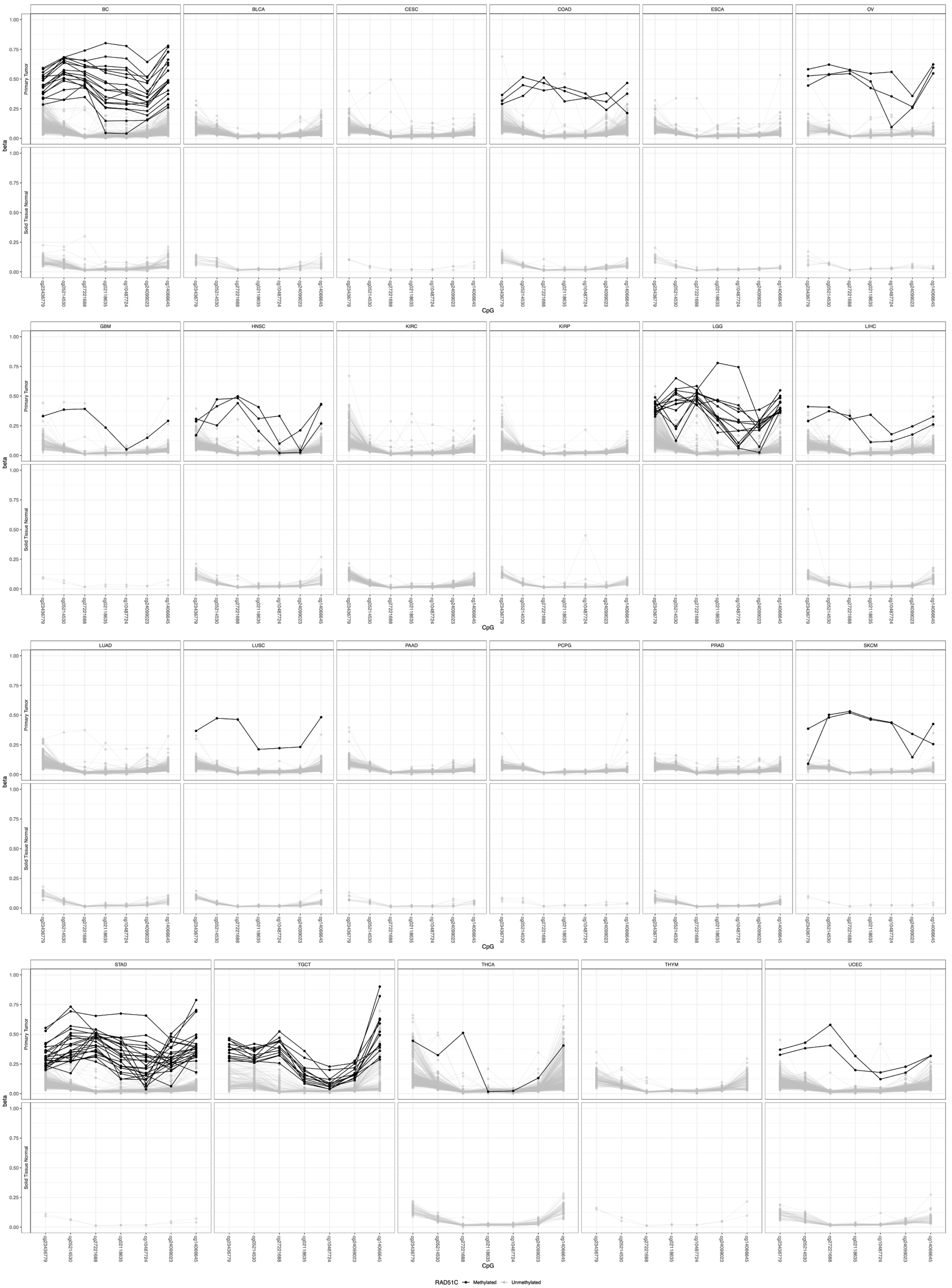

BC
BLCA
CESC
COAD
ESCA
OV
GBM
HNSC
KIRC
KIRP
LGG
LIHC
LUAD
LUSC
PAAD
PCPG
PRAD
SKCM
STAD
TGCT
THCA
THYM
UCEC
Primary Tumor
Solid Tissue Normal
beta
1.00
0.75
0.50
0.25
0.00
cg23436779
cg05214530
cg27221688
cg02118635
cg10487724
cg24099023
cg14066645
CpG
RAD51C
Methylated
Unmethylated

Figure S8. Methylation values at *RAD51C* promoter CpG probes. The plots depicting the methylation profiles of individual primary tumours and the normal samples for each analysed cancer type are shown. Individual set of dots connected by a line represent the estimated beta values from each case at the corresponding CpG (grey: predicted unmethylated, black: predicted promoter methylated). CpG probes (x-axis) are arranged in order of genomic location, however distances are not scaled by genomic coordinates. Only CpG probes selected for analysis are shown.

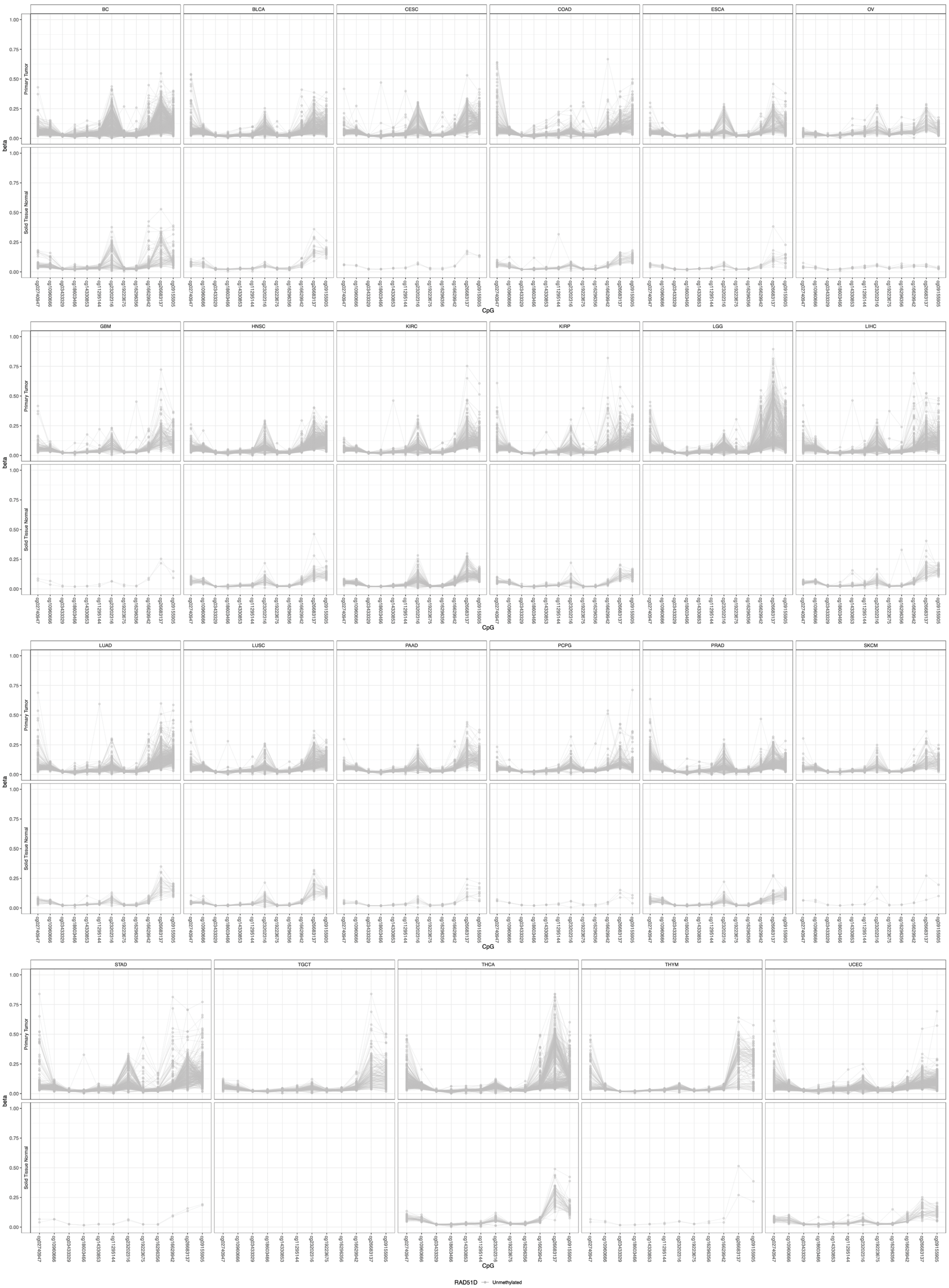
BC
BLCA
CESC
COAD
ESCA
OV
GBM
HNSC
KIRC
KIRP
LGG
LIHC
LUAD
LUSC
PAAD
PCPG
PRAD
SKCM
STAD
TGCT
THCA
THYM
UCEC
Primary Tumor
Solid Tissue Normal
beta
1.00
0.75
0.50
0.25
0.00
cg02740947
cg10960666
cg03433329
cg18603466
cg14330853
cg11295144
cg23202316
cg19228675
cg16296356
cg16629942
cg26683137
cg09155905
CpG
RAD51D
Unmethylated

Figure S9. Methylation values at *RAD51D* promoter CpG probes. The plots depicting the methylation profiles of individual primary tumours and the normal samples for each analysed cancer type are shown. Individual set of dots connected by a line represent the estimated beta values from each case at the corresponding CpG (grey: predicted unmethylated, black: predicted promoter methylated). CpG probes (x-axis) are arranged in order of genomic location, however distances are not scaled by genomic coordinates. Only CpG probes selected for analysis are shown.

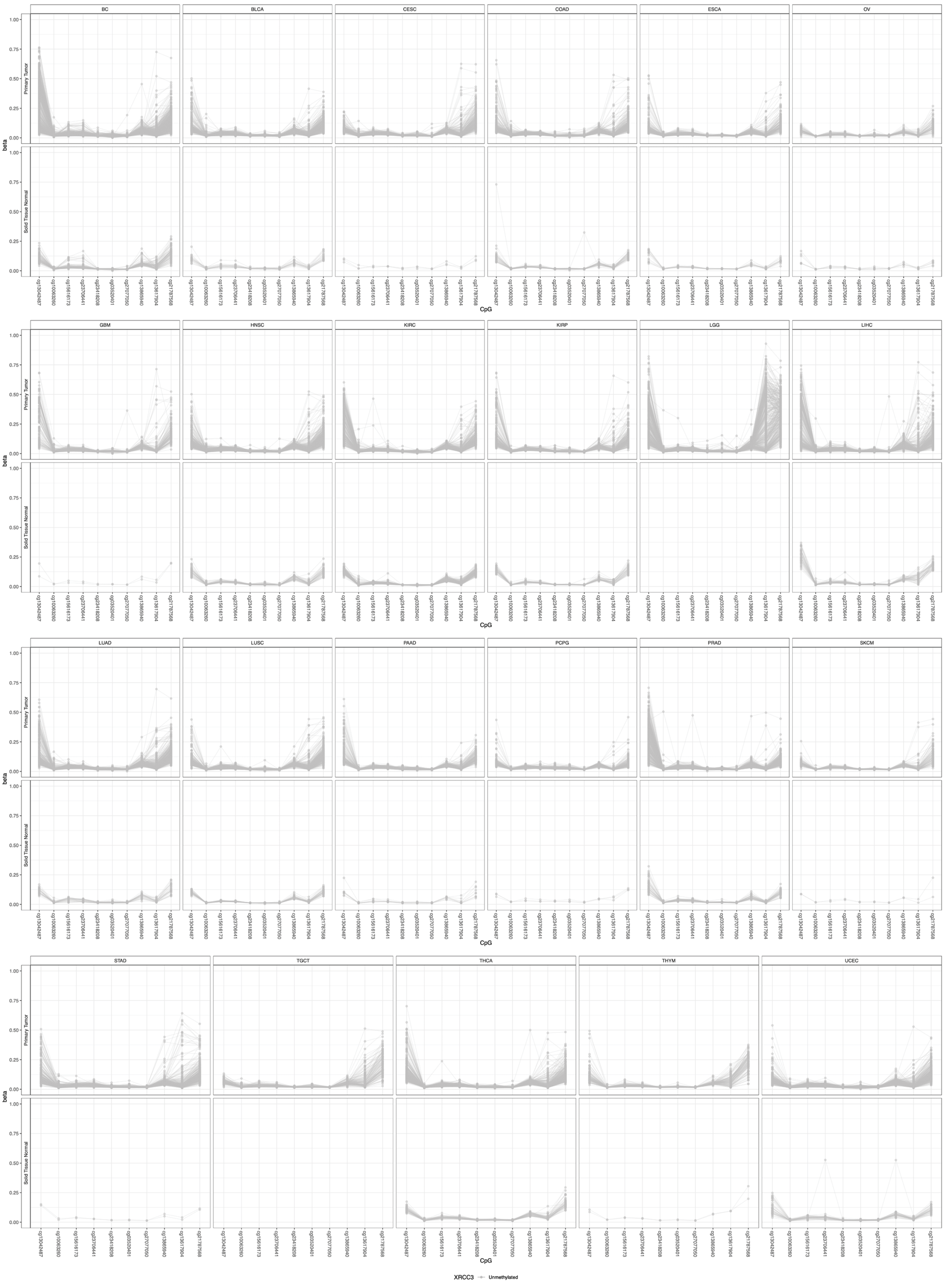
BC
BLCA
CESC
COAD
ESCA
OV
GBM
HNSC
KIRC
KIRP
LGG
LIHC
LUAD
LUSC
PAAD
PCPG
PRAD
SKCM
STAD
TGCT
THCA
THYM
UCEC
Primary Tumor
Solid Tissue Normal
beta
1.00
0.75
0.50
0.25
0.00
cg13042487
cg10063260
cg15616173
cg23706441
cg23418208
cg03520401
cg27070050
cg13865940
cg13617904
cg21787568
CpG
XRCC3
Unmethylated

Figure S10. Methylation values at *XRCC3* promoter CpG probes. The plots depicting the methylation profiles of individual primary tumours and the normal samples for each analysed cancer type are shown. Individual set of dots connected by a line represent the estimated beta values from each case at the corresponding CpG (grey: predicted unmethylated, black: predicted promoter methylated). CpG probes (x-axis) are arranged in order of genomic location, however distances are not scaled by genomic coordinates. Only CpG probes selected for analysis are shown.

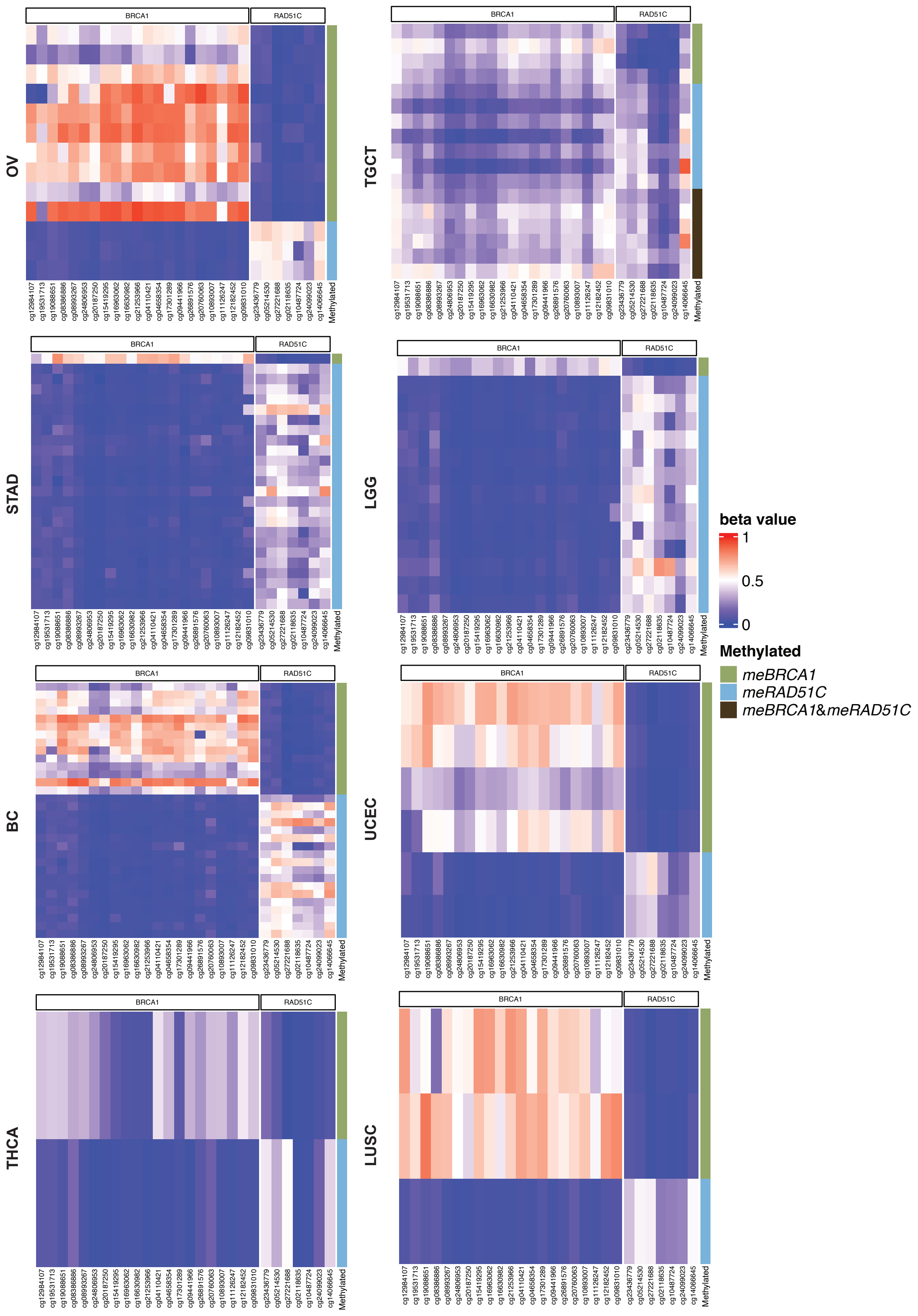

OV
TGCT
STAD
LGG
BC
UCEC
THCA
LUSC
BRCA1
RAD51C
cg12984107
cg19531713
cg19088651
cg08386886
cg08993267
cg24806953
cg20187250
cg15419295
cg16963062
cg16630982
cg21253966
cg04110421
cg04658354
cg17301289
cg09441966
cg26891576
cg20760063
cg10893007
cg11126247
cg12182452
cg09831010
cg23436779
cg05214530
cg27221688
cg02118635
cg10487724
cg24099023
cg14066645
Methylated
beta value
1
0.5
0
Methylated
meBRCA1
meRAD51C
meBRCA1&meRAD51C

Figure S11. Heatmap of beta values for CpG probes used to assess for promoter methylation status for *BRCA1* and *RAD51C* in eight cancer types with incidences of promoter methylation in both genes. Each sample is represented by row. Each column represents the beta values of CpG probes (beta value: 0-blue, 1-red) that have been ordered by chromosome location. CpG probes that locate in the promoter region and/or promoter overlapping CpG island (CGI) were selected if their median and interquartile range (IQR) of the beta value across the tumour and normal samples is less than or equal to 0.2. Using the selected probes and applying the threshold (60% of the selected probes had beta values greater than or equal to 0.25) to both *BRCA1* and *RAD51C*, the promoter methylation status assignment for each sample is as indicated on the right-hand column (*BRCA1* promtoer methylation: green; *RAD51C* promoter methylation: blue; promoter methylation identified in both gene: black). Data shown for methylated cases in high grade serous ovarian (OV; n=13), testicular germ cell (TGCT; n=17), stomach adenocarcinoma (STAD; n=25), low grade glioma (LGG; n=14), breast (BC; n=32), thyroid (THCA; n=2) and lung squamous cell carcinoma (LUSC; n=3).

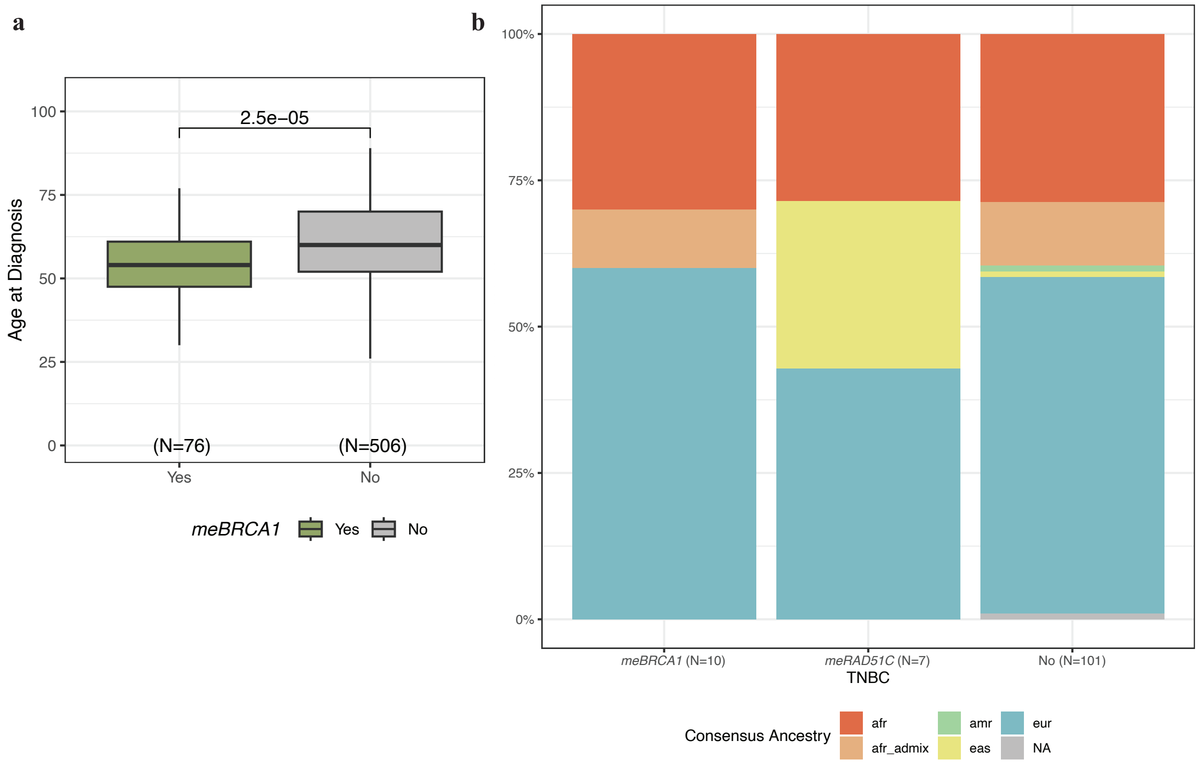

a
2.5e−05
Age at Diagnosis
100
75
50
25
0
(N=76)
(N=506)
Yes
No
meBRCA1
Yes
No
b
100%
75%
50%
25%
0%
meBRCA1 (N=10)
meRAD51C (N=7)
No (N=101)
TNBC
Consensus Ancestry
afr
afr_admix
amr
eas
eur
NA

Figure S12. Additional clinical characteristics of patients with primary tumours of high-grade serous ovarian (OV) or breast cancer (BC) exhibiting *BRCA1* or *RAD51C* promoter methylation. (a) Age distribution of TCGA OV cohort according to *BRCA1* promoter methylation status. The boxplots are composed of the median (center bar) and interquartile range (IQR), with the whiskers extending to the maximum and minimum values, but no further than 1.5 times the IQR. P-value was obtained from two-tail Wilcoxon. (b) Ancestry distribution of the triple negative breast cancer (TBNC) subset from TCGA BC. Colour of the bars indicate different ancestral origins as inferred from SNP arrays. The colour code is as follows: African (afr): orange, African admix (afr_admix): ligh orange, American (amr): green, East Asian (eas): yellow, European (eur): blue, missing information (NA): grey.

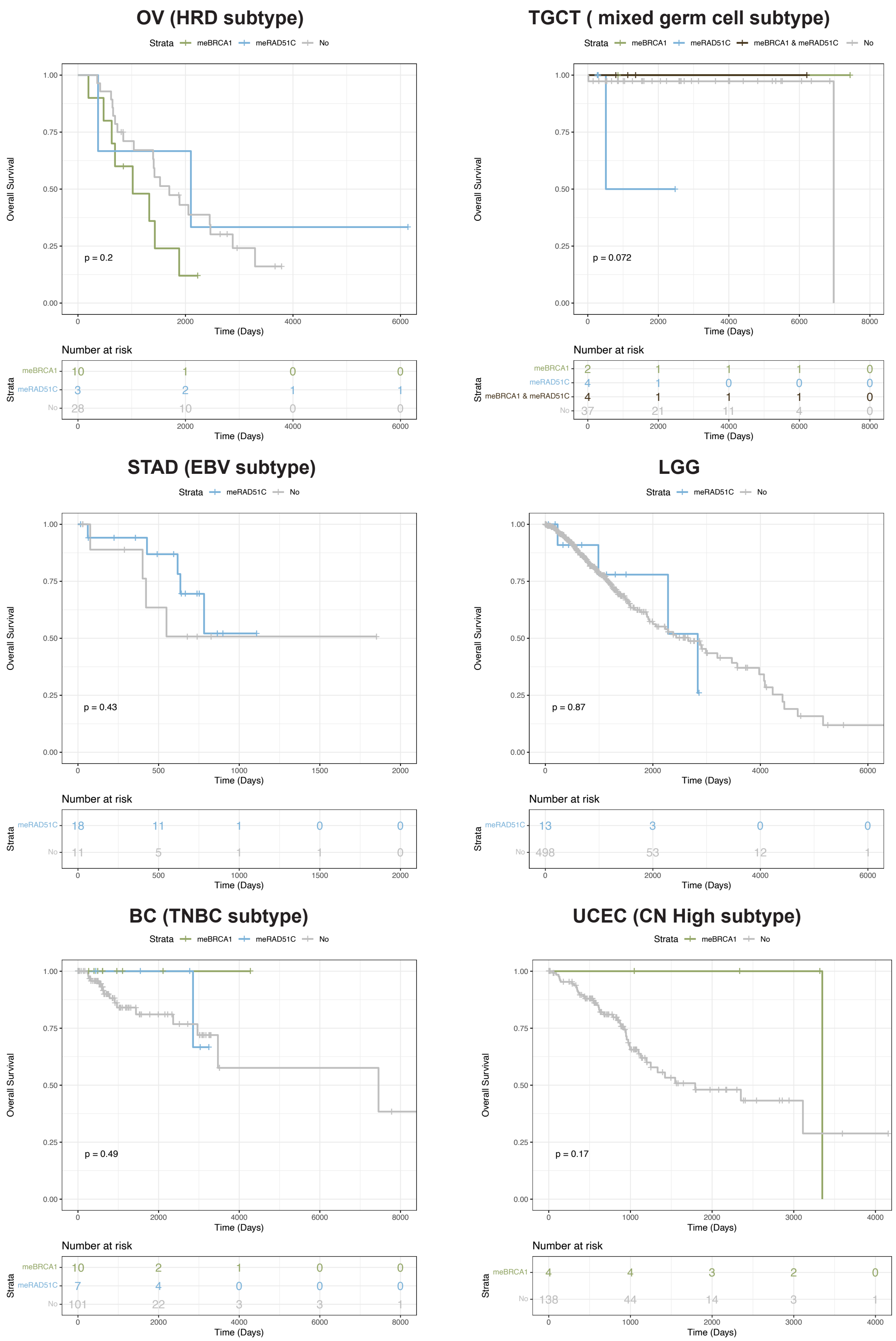

OV (HRD subtype)
Strata meBRCA1 meRAD51C No
Overall Survival
p = 0.2
Time (Days)
Number at risk
meBRCA1 10 1 0 0
meRAD51C 3 2 1 1
No 28 10 0 0
TGCT (mixed germ cell subtype)
Strata meBRCA1 meRAD51C meBRCA1 & meRAD51C No
p = 0.072
Number at risk
meBRCA1 2 1 1 1 0
meRAD51C 4 1 0 0 0
meBRCA1 & meRAD51C 4 1 1 1 0
No 37 21 11 4 0
STAD (EBV subtype)
Strata meRAD51C No
p = 0.43
Number at risk
meRAD51C 18 11 1 0 0
No 11 5 1 1 0
LGG
Strata meRAD51C No
p = 0.87
Number at risk
meRAD51C 13 3 0 0
No 498 53 12 1
BC (TNBC subtype)
Strata meBRCA1 meRAD51C No
p = 0.49
Number at risk
meBRCA1 10 2 1 0 0
meRAD51C 7 4 0 0 0
No 101 22 3 3 1
UCEC (CN High subtype)
Strata meBRCA1 No
p = 0.17
Number at risk
meBRCA1 4 4 3 2 0
No 138 44 14 3 1
Strata
Time (Days)

Figure S13. Kaplan-Meier overall survival curves of patients and their promoter methylation status in *BRCA1* and *RAD51C*. Kaplan-Meier analysis plotting the survival curve of subgroups from six different cancer types. The statistical significance was assessed using the log-rank test. Subtype of cancer with at least four cases showing promoter methylation in either *BRCA1* or *RAD51C* are included. The table below each plots detailed number at risk. Promoter methylation status is colour-coded: black (co-exist *meBRCA1* and *meRAD51C*), green (*meBRCA1*), blue (*meRAD51C*), grey (no promoter methylation detected in either gene). Abbreviation of cancer types and subtypes are: high-grade serous ovarian cancer (OV): homologous recombination deficiency (HRD) subtype; testicular germ cell tumour (TGCT); stomach adenocarcinoma (STAD): Epstein-Barr virus infected (EBV) subtype; low-grade glioma (LGG); breast cancer (BC): triple-negative breast cancer (TNBC) subtype; uterine corpus endometrial carcinoma (UCEC): copy number high (CN HIGH) subtype.

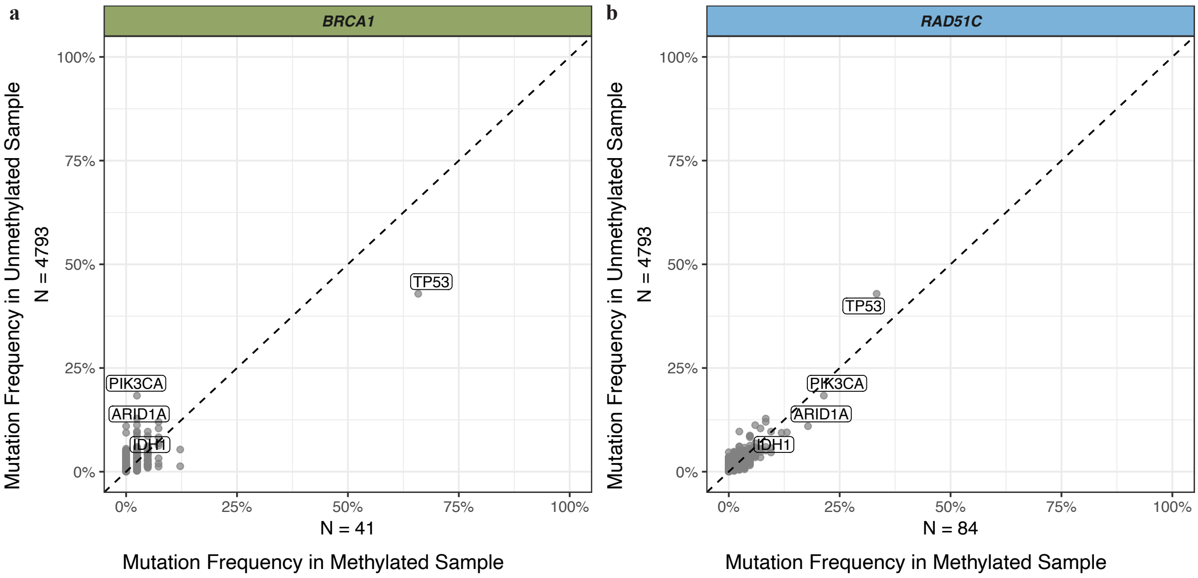

a
BRCA1
b
RAD51C
Mutation Frequency in Unmethylated Sample
N = 4793
100%
75%
50%
25%
0%
TP53
PIK3CA
ARID1A
IDH1
0%
25%
50%
75%
100%
N = 41
N = 84
Mutation Frequency in Methylated Sample

Figure S14. The frequency of somatic non-silent mutation in 1,025 cancer genes (OncoKB^{TM}) in samples with *BRCA1* or *RAD51C* promoter methylation compared to samples with no promoter methylation in either gene. Mutation frequencies across all cancer types with *BRCA1* (a) or *RAD51C* (b) promoter methylation in *TP53*, *PIK3CA*, *ARID1A* and *IDH1* are highlighted as they are relevant to either of the patterns of global hypermethylation (*PIK3CA*, *ARID1A* and *IDH1*) in stomach adenocarcinoma and low-grade glioma, or homologous recombination deficiency (HRD) in breast and ovarian cancer (*TP53*).

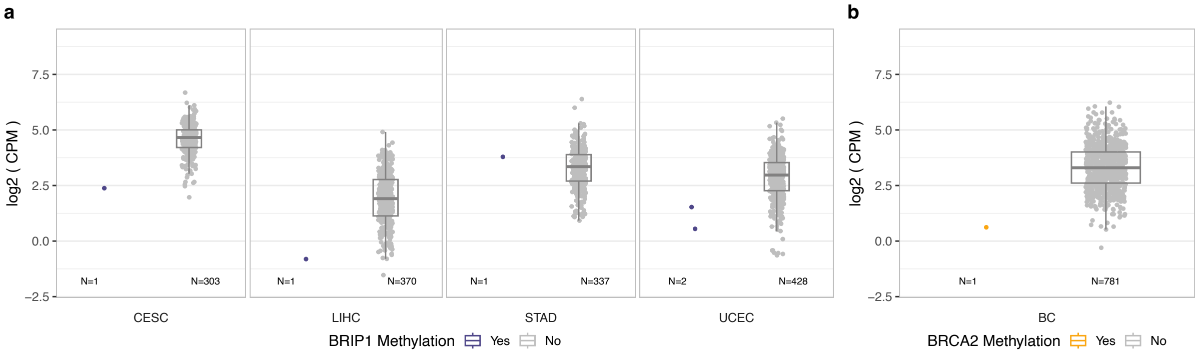

a
b
log2 (CPM)
7.5
5.0
2.5
0.0
−2.5
N=1
N=303
N=1
N=370
N=1
N=337
N=2
N=428
N=1
N=781
CESC
LIHC
STAD
UCEC
BC
BRIP1 Methylation
Yes
No
BRCA2 Methylation
Yes
No

Figure S15. *BRIP1* and *BRCA2* promoter methylation associated gene expression.

Gene expression of *BRIP1* (a) and *BRCA2* (b) in samples grouped by the presence of promoter methylation. Each box plot is a cancer type with normalised mRNA gene expression (log2 CPM) on y-axis and samples on x-axis grouped by gene promoter methylation status. The boxplots are composed of the median (center bar) and IQR, with the whiskers extending to the maxima and minima, but no further than 1.5 x IQR. Outliers, defined as values beyond 1.5 * IQR, are not shown in the boxplot. All data points are depicted with dots overlaid on the boxplot. Abbreviations: cervical squamous cell carcinoma (CESC), hepatocellular carcinoma (LIHC), stomach adenocarcinoma (STAD), uterine corpus endometrial carcinoma (UCEC), breast cancer (BC).

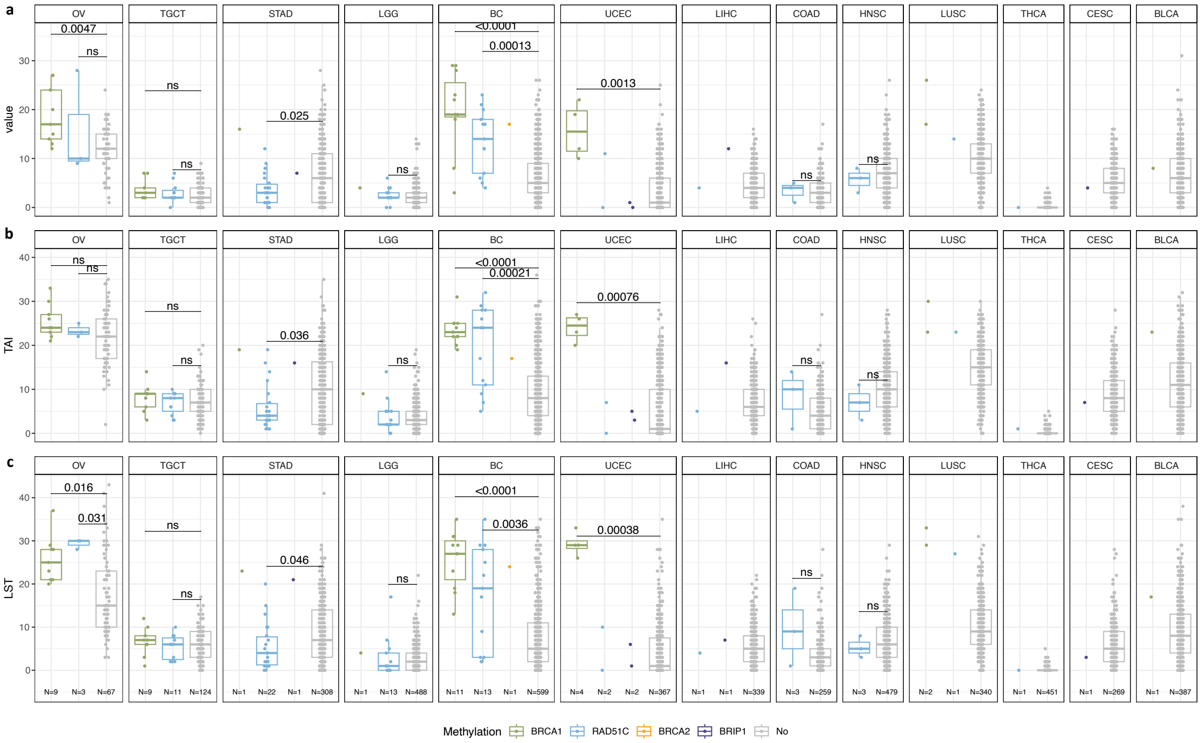
a
b
c
OV
TGCT
STAD
LGG
BC
UCEC
LIHC
COAD
HNSC
LUSC
THCA
CESC
BLCA
value
TAI
LST
Methylation
BRCA1
RAD51C
BRCA2
BRIP1
No

Figure S16. Components of homologous recombination deficiency (HRD) scores in cases with promoter methylation of HRR genes. Cancer types with case showing methylation in any of the homologous recombination repair (HRR) genes are included. This plot shows the number of the loss of heterozygosity (LOH) (a), telomeric allelic imbalance (TAI) (b) and large-scale state transitions (LST) events (c) among the cases grouped by their promoter methylation status of these gene. Each boxplot describes the distribution of LOH, TAI, LST in cancer types with more than three promoter methylation incidences of one of the gene. The boxplots are composed of the median (center bar) and interquartile range (IQR), with the whiskers extending to the maximum and minimum values, but no further than 1.5 times the IQR. Outliers, defined as values beyond 1.5 times the IQR, are not shown with the boxplot. All data points are superimposed with dots on the boxplot. To compare the distribution of each component, in cancer types with at least three promoter methylation samples, a two-tailed Wilcoxon signed-rank test with Benjamini-Hochberg correction was conducted. Abbreviation of cancer types are: high-grade serous ovarian cancer (OV), testicular germ cell tumour (TGCT), stomach adenocarcinoma (STAD), low-grade glioma (LGG), breast cancer (BC), uterine corpus endometrial carcinoma (UCEC), glioblastoma (GBM), liver hepatocellular carcinoma (LIHC), colon adenocarcinoma (COAD), thyroid cancer (THCA), cervical squamous cell carcinoma (CESC) and bladder urothelial carcinoma (BLCA).

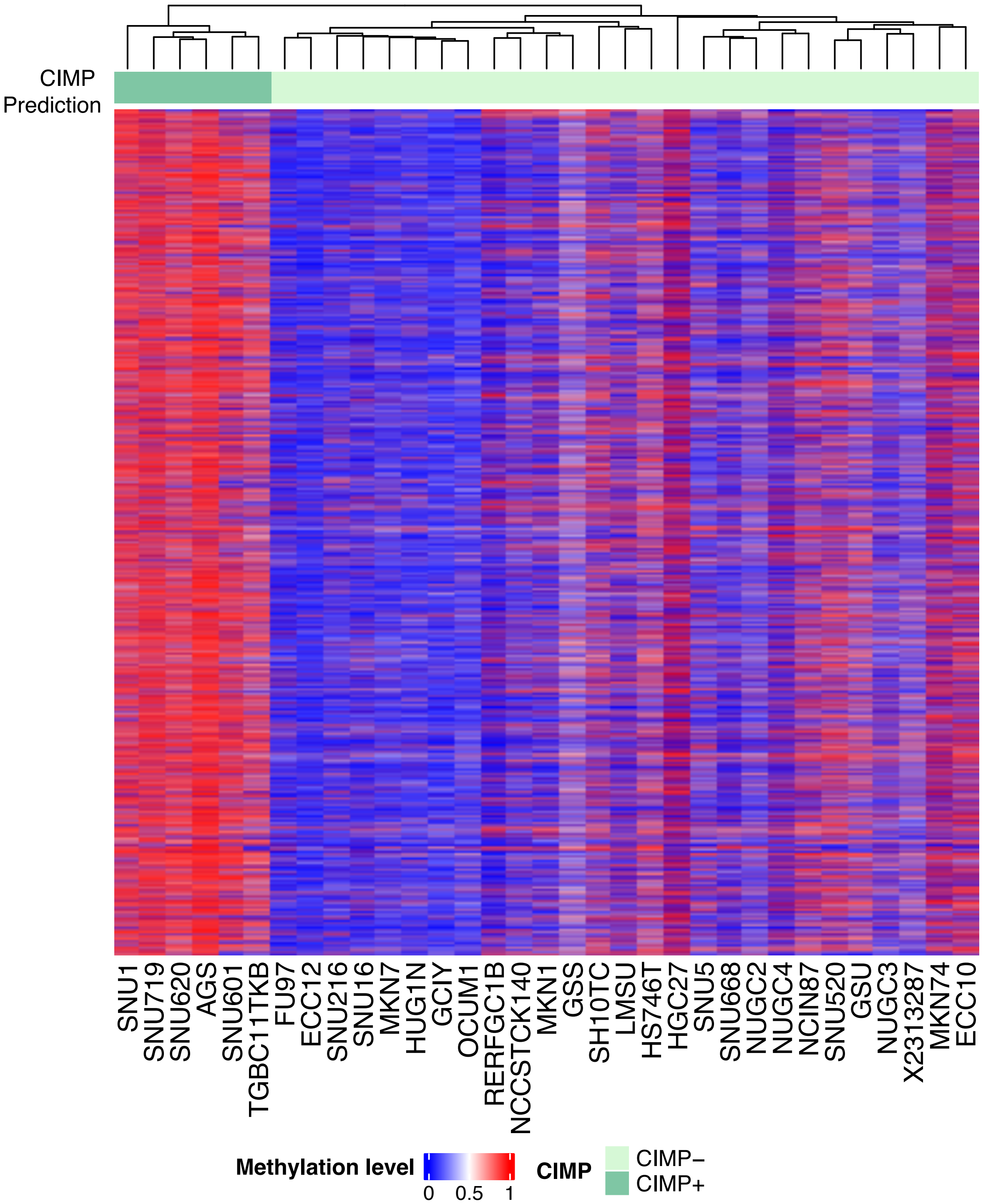

CIMP
Prediction
SNU1
SNU719
SNU620
AGS
SNU601
TGBC11TKB
FU97
ECC12
SNU216
SNU16
MKN7
HUG1N
GCIY
OCUM1
RERFGC1B
NCCSTCK140
MKN1
GSS
SH10TC
LMSU
HS746T
HGC27
SNU5
SNU668
NUGC2
NUGC4
NCIN87
SNU520
GSU
NUGC3
X2313287
MKN74
ECC10
Methylation level
0
0.5
1
CIMP
CIMP−
CIMP+

Figure S17. Clustering of global methylation in 33 DepMap stomach cancer cell-lines. Hierarchical clustering of 31,483 highly variable CpG sites on autosomal chromosomes. Bases on hierarchical clustering, 33 cell-lines are categorised into groups with global high methylation (CIMP+; green) and global low methylation (CIMP-; light green).
